# MAGE-A Cancer/Testis Antigens Inhibit MDM2 Ubiquitylation Function and Promote Increased Levels of MDM4

**DOI:** 10.1371/journal.pone.0127713

**Published:** 2015-05-22

**Authors:** Lynnette Marcar, Bianca Ihrig, John Hourihan, Susan E. Bray, Philip R. Quinlan, Lee B. Jordan, Alastair M. Thompson, Ted R. Hupp, David W. Meek

**Affiliations:** 1 Division of Cancer Research, University of Dundee, Clinical Research Centre and Jacqui Wood Cancer Centre, Ninewells Hospital, James Arrott Drive, Dundee, United Kingdom; 2 School of Veterinary Medicine and Science, University of Nottingham, Sutton Bonington Campus, Leicestershire, United Kingdom; 3 School of Computer Science, University of Nottingham, Jubilee Campus, Nottingham, United Kingdom; 4 Advanced Data Analysis Centre, University of Nottingham, Nottingham, United Kingdom; 5 M. D. Anderson Cancer Center, University of Texas, 1400 Pressler Drive, Unit 1484, Houston, United States of America; 6 p53 Signal Transduction Laboratory, Edinburgh Cancer Research UK Centre, The University of Edinburgh, Crewe Road South, Edinburgh, United Kingdom; Georgia Regents University, UNITED STATES

## Abstract

Melanoma antigen A (MAGE-A) proteins comprise a structurally and biochemically similar sub-family of Cancer/Testis antigens that are expressed in many cancer types and are thought to contribute actively to malignancy. MAGE-A proteins are established regulators of certain cancer-associated transcription factors, including p53, and are activators of several RING finger-dependent ubiquitin E3 ligases. Here, we show that MAGE-A2 associates with MDM2, a ubiquitin E3 ligase that mediates ubiquitylation of more than 20 substrates including mainly p53, MDM2 itself, and MDM4, a potent p53 inhibitor and MDM2 partner that is structurally related to MDM2. We find that MAGE-A2 interacts with MDM2 via the N-terminal p53-binding pocket and the RING finger domain of MDM2 that is required for homo/hetero-dimerization and for E2 ligase interaction. Consistent with these data, we show that MAGE-A2 is a potent inhibitor of the E3 ubiquitin ligase activity of MDM2, yet it does not have any significant effect on p53 turnover mediated by MDM2. Strikingly, however, increased MAGE-A2 expression leads to reduced ubiquitylation and increased levels of MDM4. Similarly, silencing of endogenous MAGE-A expression diminishes MDM4 levels in a manner that can be rescued by the proteasomal inhibitor, bortezomid, and permits increased MDM2/MDM4 association. These data suggest that MAGE-A proteins can: (i) uncouple the ubiquitin ligase and degradation functions of MDM2; (ii) act as potent inhibitors of E3 ligase function; and (iii) regulate the turnover of MDM4. We also find an association between the presence of MAGE-A and increased MDM4 levels in primary breast cancer, suggesting that MAGE-A-dependent control of MDM4 levels has relevance to cancer clinically.

## Introduction

Melanoma-associated antigens (MAGE) were initially discovered as cancer-associated antigens in melanoma patients [1] and are now known to comprise a super-family (encompassing several sub-families) of more than 60 genes in humans [2,3]. MAGEs are subdivided into two groups, MAGE-I and MAGE-II, based on the chromosomal locations of the genes and the tissue distribution of their products [2,3]. MAGE-I proteins (comprising sub-families MAGE-A, -B and -C) are members of the broader family of Cancer/Testis (CT) Antigens which are physiologically expressed mainly in germ cells, but are aberrantly expressed, mainly through epigenetic reprogramming, in a wide range of cancers including those of breast, ovary, lung, and bladder [3,4,5,6]. MAGE-II proteins are more widely expressed and are not normally associated with cancer.

Members of the MAGE-A sub-family show striking structural and functional similarity to each other [3]. Expression of MAGE-A is observed mainly in cancers that have acquired malignant phenotypes such as invasiveness or metastasis, and correlates with poor prognosis [3]. Curiously, MAGE-A genes are frequently co-expressed, with most cancers showing the presence of at least two or more of these antigens. The levels of expression can also vary enormously and may consequently affect the biological fate of cells expressing these proteins. Consistent with a cancer-promoting role, MAGE-A expression stimulates cell cycle progression, migration rate and invasiveness of cultured cells *in vitro* and can promote increases in tumor size and in the number and size of metastatic foci in animal models [7,8]. Collectively, these data support the idea that MAGE-A expression may contribute towards malignancy.

The normal function(s) of the MAGE-A family remains unknown but growing evidence suggests these proteins modulate key transcription factors such as SKIP, p300, p160(TIF2) androgen receptor ER-alpha, and the p53 tumour suppressor [3,7,9,10,11,12,13,14,15]. Through interaction with these proteins, MAGE-A (and other MAGE I proteins) can regulate transcriptional events by various mechanisms such as the recruitment and targeting of histone deacetylase (HDAC) activity to SKIP or p53 [11,13,16], promoting interaction of the androgen receptor with p160 and other co-activator proteins [14], or the coupling of the co-repressor, KAP1/TRIM28, to KRAB domain zinc finger (KZNF) transcription factors [8,17,18]. In addition to these effects, recent evidence indicates that a range of MAGE proteins (both types I and II) can promote ubiquitylation by acting as activators of RING (really interesting new gene) finger-type E3 ubiquitin ligases [8,17,19]. For example, MAGE-G1 can stimulate the ubiquitin ligase activity of NSE1. Similarly, the interaction of MAGE-C2 with the KAP1 (TRIM28) corepressor protein can stimulate KAP1-dependent turnover of the p53 tumor suppressor independently of the major p53 regulator, MDM2 [17]. This unifying theme of MAGE proteins as modulators of RING finger ligases is underpinned by the observation that nine novel MAGE-I binding partners identified by TAP-tag/mass spectrometry analysis are RING finger proteins [17]. Within the MAGE-A family, current biochemical evidence suggests a high degree of functional redundancy, for example in regulating p53 function [12,13]. On the other hand, recent publications indicate that there may be a high degree of specificity in MAGE/partner interactions, at least for some partners [14,15,17].

p53 acts mainly as a transcription factor that eliminates cancer cells by coordinating changes in gene expression, leading to cell cycle arrest, senescence or apoptosis [20]. p53 is regulated primarily through its interaction with MDM2, a RING finger-type ubiquitin E3 ligase. MDM2 and p53 operate within a feedback loop in which p53 stimulates *MDM2* expression, leading to down-regulation of p53 levels by MDM2. Induction and activation of p53 by various stress stimuli is achieved through different but overlapping mechanisms that principally uncouple the p53/MDM2 interaction [21]. The major features of the MDM2 protein are: an N-terminal p53 binding domain in which the N-terminus of p53 interacts with a hydrophobic pocket on MDM2; nuclear export and import sequences which mediate nucleo-cytoplasmic shuttling of both MDM2 and p53; a highly acidic and highly phosphorylated central region that is crucial for p53 degradation; and a C-terminal RING finger which is required for both the ubiquitylation and degradation of p53. These domains underpin several biochemical functions in MDM2 which act sequentially and cooperatively to down-regulate p53 levels: these include mainly ubiquitylation, nucleo-cytoplasmic transport, and targeting of p53 to the proteasome for degradation. Additionally, these biochemical functions can be regulated *independently* of each other. For example, whereas multi-site phosphorylation of the acidic central domain of MDM2 has no detectable effect on its ability to mediate ubiquitylation of p53, these modifications play a crucial role in promoting the turnover of p53 by stimulating the association of MDM2 with several components of the proteasome [22,23]. Although capable of mediating interaction with the proteasome, the precise mechanism(s) by which MDM2 drives p53 degradation is incompletely understood.

MDM4 (also known as MDMX or HDMX) is a defective ubiquitin ligase that is structurally related to MDM2 and inhibits p53 through overlapping mechanisms [24,25]. Firstly, it is a stimulatory partner of MDM2 [26] that is itself regulated by MDM2-mediated ubiquitylation [27,28,29]. Moreover, as with MDM2, p53 binds to MDM4 principally through an N-terminal hydrophobic pocket thereby sterically preventing the interaction of the p53 transactivation domain (TAD1) with the transcriptional machinery and thereby down-regulating p53-mediated transactivation [30].

p53 function is lost during the development of a high proportion of cancers of different types through mutation of the *TP53* gene (encoding p53). In some cancers, however, the *TP53* mutation frequency is lower but other mechanisms, such as altered expression or mutation of p53 regulators (e.g. MDM2, MDM4, ARF) are thought to eliminate p53 function [31]. We and others have shown that different members of the MAGE-A family, all of which show striking sequence similarity, act in a similar manner and are potent inhibitors of p53-mediated transcription and apoptosis [8,12,13]. Two of these studies show that MAGE-A acts, at least in part, by binding to the core (site-specific DNA binding) domain of p53 and regulating its downstream transcription function [12,13]. MAGE-A can also regulate p53 acetylation (activation) through HDAC3 recruitment and interaction with PML nuclear bodies [13,16]. Through these mechanisms MAGE-A can confer resistance to drugs that act through the p53 pathway [13]. Interestingly, some [17,32], but not all [12,13], studies suggest that MAGE proteins can regulate p53 levels, for example through interaction with E3 ligases such as KAP1 (TRIM28) [17]. However, this issue has not been resolved. Moreover, given the now established role of MAGE-A proteins as regulators of RING finger E3 ligases it has not been demonstrated whether these proteins can impact on the role of MDM2.

In the present study we show that endogenous MAGE-A proteins and, via ectopic expression, MAGE-A2, associate with MDM2 *independently* of p53. This observation prompted us to explore the interaction between these two proteins in greater depth with a view to understanding the functional significance of their association. Our data indicate that, in addition to regulating p53 function directly, MAGE-A can selectively influence the levels of MDM4 through interaction with MDM2, and support the idea that this family of proteins regulates p53 function through several independent but complementary mechanistic events. We also establish that MAGE-A proteins, while acting as stimulators of some RING finger type proteins [17], can also behave as a potent inhibitor of the MDM2 RING finger type E3 ubiquitin ligase.

## Materials and Methods

### Cell lines, transfections, and plasmids

U2OS (human osteosarcoma-derived) cells and H1299 (human lung carcinoma-derived) cells were obtained from the Cancer Research UK depository. The cells were tested for p53- and MAGE-A status by western blotting. Transfections were carried out using Lipofectamine reagent (Invitrogen) as instructed by the manufacturer. Plasmids used for the expression of human cDNAs were derivatives of pcDNA3 and were as follows (with catalogue numbers in parentheses): wild type p53 in pCDNA3 (DWM715), HA-tagged MAGE-A2 in pCDNA3 (DWM1574), human wild type MDM2 (under CMV promoter; DWM1127), MDM2-C464A (DWM1214), HA-tagged (N-terminus) human MDM4 (1318), His_6_ tagged ubiquitin (DWM1432). Glutathione S-transferase (GST)-fusion proteins were encoded in derivatives of the vector, pGEX-4T. These included a set of previously published MDM2 “mini-proteins” where overlapping domains of MDM2 are fused to GST [33,34] and were as follows: GST alone (DWM223), GST-MP1 (DWM1074), GST-MP2 (DWM1093), GST-MP3 (DWM1076), GST-MP4 (DWM1077).

### Antibodies and Western blot analysis

SDS-PAGE and western blotting was carried out using standard conditions. Antibodies used were as follows: 6C1 (pan MAGE-A), and H164 (p21) were obtained from Santa Cruz Biotechnology. DO1 (p53), 4B2 (MDM2) and SMP14 (MDM2) were from Moravian Biotechnology. CM1 was a kind gift from Prof. Sir D. Lane. Anti-MDM4 antibodies 8C6 and BL1258 were obtained from Millipore and from Bethyl Laboratories respectively. 12CA5 (HA-tag) was from Cancer Research UK and 20–33 (actin) was from Sigma-Aldrich. HRP (horseradish peroxidase)-conjugated rabbit anti-mouse and goat anti-rabbit secondary antibodies were purchased from Dako and Bio-Rad, respectively. Proteins were detected by enhanced chemiluminescence according to the manufacturer's instructions (Pierce Biotechnology, Inc.).

### GST-pull-down Experiment

GST-tagged MDM2 fusion proteins [33,34] (or GST alone as control) were bound to GSH (Glutathione Sepharose 4B) beads (Amersham) and subsequently incubated with ^35^S-labelled MAGE-A2 (prepared by *in vitro* transcription/translated TNT T7 System (Promega)). After extensively washing the beads, immobilized proteins were eluted in 2 X SDS sample buffer and detected by western blotting.

### *In vitro* pull-down assays using biotinylated peptides

Biotinylated peptides were purchased from Mimotopes and generally comprised the following format: "Biotin-SGSG-PEPTIDE-amide". Peptides representing the N- and C-termini were, respectively: "Amine-PEPTIDE-GSG-Biocytin amide" and "Biotin-SGSG-PEPTIDE-acid". Streptavidin-agarose beads (Sigma) were washed in PBS containing 0.1% (v/v) of Tween-20 prior to the addition of 10 μg of biotinylated peptide. Beads were incubated overnight at 4°C and subsequently washed with the same buffer. ^35^S-labeled MAGE-A2 (prepared by *in vitro* transcription/translation TNT T7 System (Promega)) was added to the beads and incubated for 2 hours at 4°C. Beads were subsequently washed 4 times in PBS/Tween-20 and resuspended in SDS-PAGE sample buffer. Co-precipitating protein was resolved by SDS-PAGE and detected by fluorography.

### Immunoprecipitation

Cells were lysed on ice for 30 min in IP buffer (50mM Tris-HCl [pH 7.5], 150mM NaCl, 1% (v/v) NP-40, 2mM EDTA and protease inhibitor (Roche)), centrifuged for 10 min at 13,000 x g and supernatant removed for analysis. The lysate was then pre-cleared with 50 μl slurry of protein G beads (Sigma) for 30 min at 4°C, centrifuged for 3 min at 2,000 x g and the beads were discarded. A total of 1–2 μg of protein was incubated with 2 μg/ml of antibody overnight at 4°C and immunoprecipitated the following day with 50 μl slurry of protein G beads (Sigma) for 2 hours at 4°C. Precipitates were washed three times for 5 min in IP buffer at 4°C before addition of SDS—gel-loading buffer and immunoprecipitated proteins were detected by western blot.

### Ubiquitylation assay

The ubiquitylation assay relies on the transfection of cells with a plasmid encoding His-tagged ubiquitin followed by the capture and purification of ubiquitylated proteins from cell extracts using nickel-agarose beads. This assay has been described in significant detail elsewhere [35]. The purified ubiquitylated proteins were analyzed by western blotting.

### Gene silencing using siRNA

MAGE-A expression was silenced using two independent, previously published siRNA oligonucleotides [12,13]: MAGE-A siRNA(1) [13] and MAGE-A siRNA(2) [12] are targeted against two different sequences common to each of the Mage-A family members except Mage-A10 and are as follows:

MAGE-A Oligo 1 [13] 5’-AACCAGCUAUGUGAAAGUC-3’

MAGE-A Oligo 2 [12] 5’-UCACAAAGGCAGAAAUGCU-3’

Non-silencing Oligo (control) 5’-CAGUCGCGUUUGCGACUGG-3’

The siRNA oligonucleotides were manufactured by Dharmacon and were introduced into cells by reverse transfection using RNAiMAX (Invitrogen) according to the manufacturer's instructions. Cells were harvested 48 h after transfection.

### Patients, Consent and Immunohistochemistry (IHC)

The cohort used in this study (TMA24) has been reported previously [36,37] and comprises primary, previously untreated breast cancer from 225 unselected pre- and post-menopausal women (aged 28–89; median 62 years) treated at Tayside University Hospitals, Scotland from 1997 to 2002. Acquisition of samples and the preparation of the tumour microarray (TMA) have been detailed elsewhere [36,37]. The study received ethical approval from both the Tayside Research and Ethics Committee (Ref. 07/S1402/90) and the Tayside Tissue Bank Committee (Ref. TR000236). Generic, written informed consent was obtained from each patient prior to tissue acquisition and before surgery was carried out, a process approved by the Research Ethics Committee, for use of excess tissue not required for diagnostic purposes and for venous blood draw to be used in research.

Sections from the TMA block (nominally 4 microns thick) were prepared and processed as described previously [37]. Antibodies specific for proteins used in the study were: 6C1 (pan-MAGE-A); and BL1258 (MDM4).

TMA scoring for MAGE-A and MDM4 was carried out by a specialist breast pathologist (LBJ) using the Quickscore method [38] for the intensity and proportion of cells stained with the appropriate antibody.

## Results

### MAGE-A proteins interact with MDM2 in cultured cells

**Fig 1:**
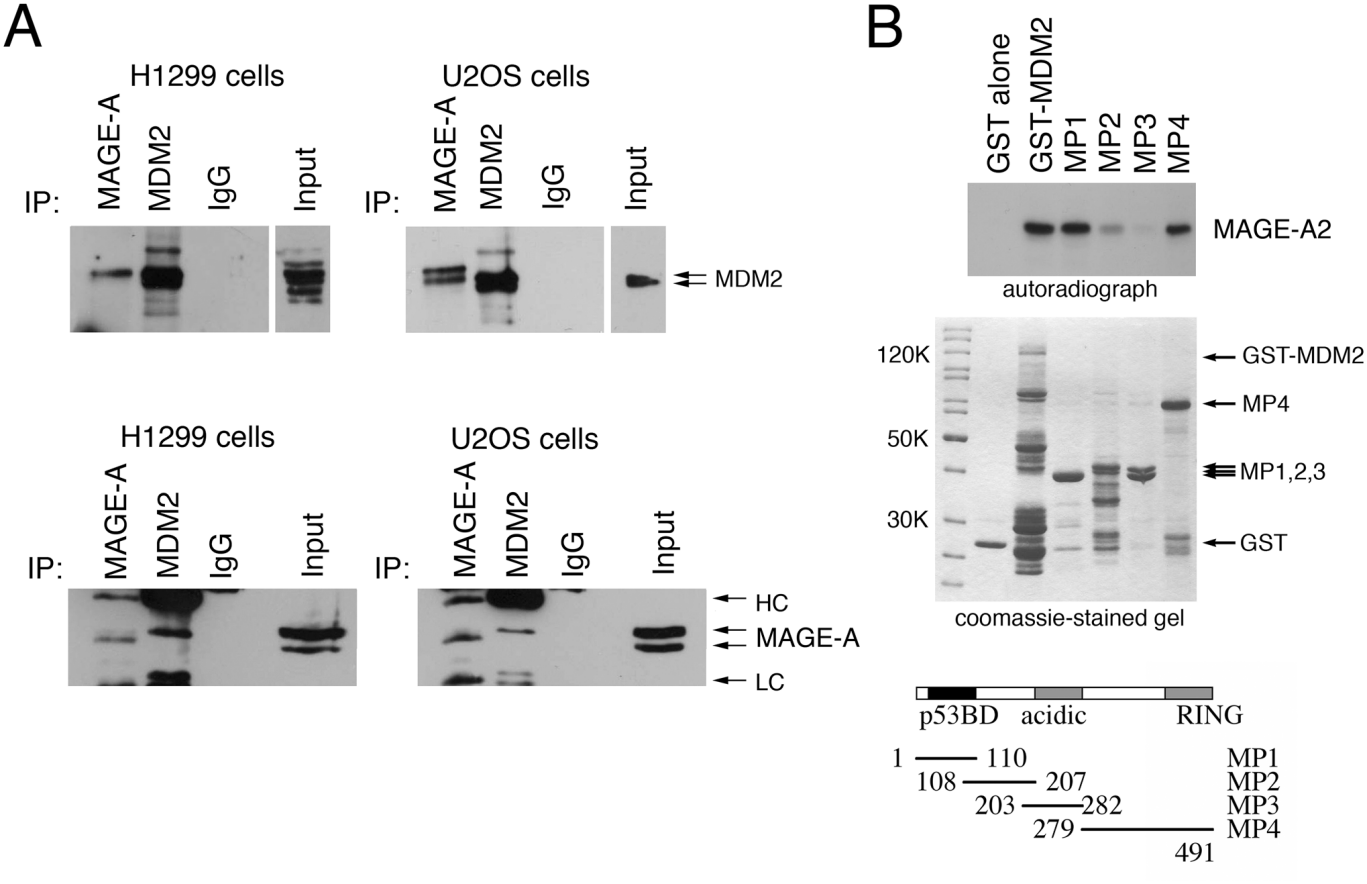
MAGE-A proteins associate with MDM2 *in vitro* and in cultured cells. (A) H1299 cells (left hand panels) or U2OS cells (right hand panels) were lysed and immunoprecipitation was carried out using antibodies against MAGE-A (6C1), MDM2 (SMP14 plus 4B2) or, as control, a non-specific murine IgG. The blots were probed for the presence of MDM2 (top panels) or MAGE-A (bottom panels). The positions of antibody heavy chains (HC) and light chains (LC) are indicated in the lower panels. (B) GST pull-down assays were performed in which ^35^S-radiolabelled MAGE-A2 was captured on glutathione sepharose 4B beads using GST linked to full length MDM2 or to four mini-proteins (termed MP1, -2, -3 and -4) representing overlapping regions of MDM2 [33,34]. The co-precipitating MAGE-A2 was detected by SDS-PAGE followed by fluorography (upper panel). The middle panel shows the presence of the GST-MDM2 proteins following binding to the glutathione beads. The bottom panel is a schematic showing full length MDM2 together with the overlapping regions encompassed within the mini-proteins. The data are representative of three independent experiments.

To determine whether MAGE-A proteins interact directly with MDM2 independently of p53, co-immunoprecipitation of endogenously expressed proteins was carried out using H1299 cells (which do not express p53). As a control, the experiments were also done in wild type p53-expressing U2OS cells which we and others have used previously for MAGE-A/p53 interaction studies [12,13,16]; both of these lines express several detectable members of the MAGE-A family (S1 Fig). Consistent with this observation the pan-MAGE-A antibody, 6C1, detected several closely migrating bands corresponding to various MAGE-A family members in the cell extracts (Fig 1A, lower panel). Immunoprecipitation of MAGE-A using 6C1 resulted in the co-immunoprecipitation of MDM2 (Fig 1A, upper panel). In a reciprocal analysis, MAGE-A proteins were found to be present in immunoprecipitates of MDM2 from the same extracts using the antibodies, 4B2 and SMP14 (lower panel). Co-immunoprecipitation was also conducted using U2OS cells (which express wild type p53) with similar findings. The slower-migrating MDM2 proteins seen to co-immunoprecipitate with MAGE-A in both cell lines suggest that MAGE-A proteins may preferentially interact with a post-translationally modified form(s) of MDM2. Similarly, slower-migrating bands of MAGE-A could suggest that modified MAGE-A proteins preferentially associate with MDM2 or, alternatively, that MDM2 associates preferentially with specific MAGE-A family members. We do not have definitive answers to these points at present. MAGE-A proteins were not detectable in MDM4 immunoprecipitates nor, reciprocally, did MDM4 co-immunoprecipitate when the anti-MAGE-A antibody was used (data not shown). These data imply specificity with the MAGE-A/MDM2 interaction. In summary, these data indicate that MDM2 and MAGE-A associate in a cellular context.

### MAGE-A proteins interact with MDM2 *in vitro*

To determine whether MDM2 and MAGE-A interact *in vitro*, GST pull-down experiments were carried out using a series of GST-fusion proteins comprising GST-full length human MDM2 together with a previously published series of GST-fusion proteins linked to overlapping domains of MDM2 [33,34]. The data (Fig 1B) confirm that ^35^S-labelled *in vitro*-translated MAGE-A2 interacts with MDM2 and not with the GST portion of the fusion protein in pull-down analysis. (MAGE-A2 was selected for this analysis because it is a well-characterised regulator of p53 pathway that performs equally well when compared with other MAGE-A proteins (-A1 and -A6) in their ability to block p53 function [12,13].) The data also show that MAGE-A2 interacts strongly with the N-terminal 110 amino acids of MDM2 and the C-terminal region comprising amino acids 279–491. There is a weak interaction with MDM2 amino acids 108–207 but very little association with amino acids 203–282 comprising mainly the important acidic domain.

**Fig 2:**
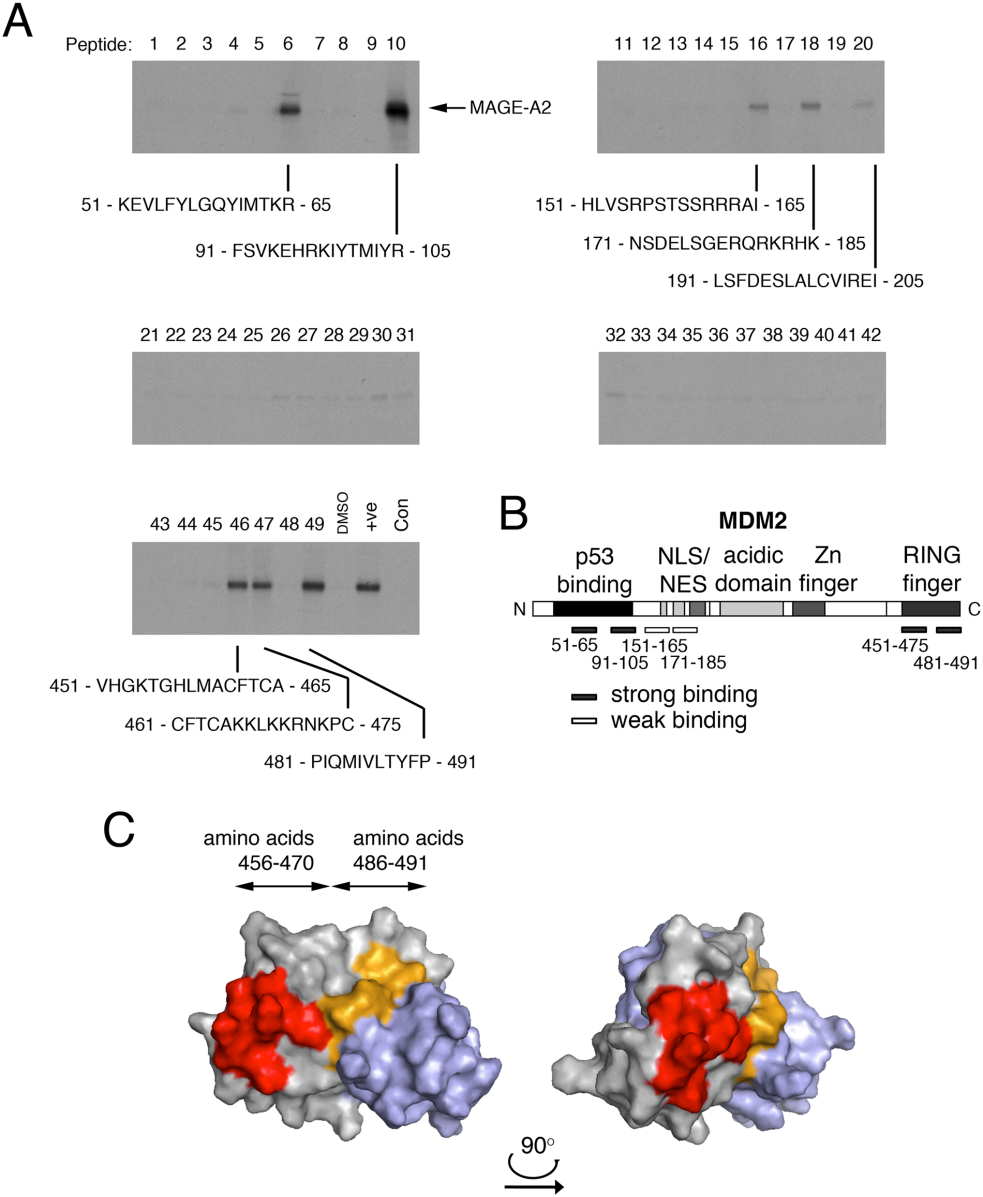
MAGE-A2 interacts with specific sites in MDM2. (A) Pepscan assays (pull-down experiments) were conducted in which a series of 15-mer biotinylated peptides (numbered 1–49) overlapping by 5 or more amino acids and representing the entire MDM2 amino acid sequence were coupled to streptavidin-sepharose beads and used to capture ^35^S-labelled, in vitro—translated MAGE-A2. The co-precipitating MAGE-A2 was detected by SDS-PAGE followed by fluorography. The control pull-down (bottom left hand panel) was a peptide representing the BoxIV/V region of p53 which we previously established binds very tightly to MAGE-A2 [12]. (B) Schematic showing the regions represented by the interacting peptides in the pull-down assay. Strong and weak binding sites are indicated in the context of important functional domains within the MDM2 protein. The data are representative of three independent experiments. (C) The model shows a dimer of MDM2 RING fingers, based on the published 3D structure (Protein Data Bank accession number: 2HDP). The two identical subunits in the dimer are represented in grey and light blue respectively. The image on the right hand side was obtained by rotating the 3D structure by approximately 90° to the right in the horizontal plane. The location of the MAGE-A2 binding peptides are shown on one subunit: amino acids 456–470 are represented in red while amino acids 486–491 are shown in light orange.

To explore further the site(s) of interaction, the ability of MDM2 amino acid sequences to co-precipitate ^35^S-labelled *in vitro*-translated MAGE-A2 was measured in pepscan assays. These assays used a respective series of 15-mer biotyinylated peptides bound to streptavidin-beads, each peptide overlapping with the next in series by 5 or more amino acids, and encompassing respectively the entire human MDM2 amino acid sequence (S2 Fig). We previously used a similar approach to determine the sites of interaction of MAGE-A2 in p53 [12]. The data from this analysis identify several peptides as interacting with MAGE-A2 (Fig 2A and summarised in Fig 2B). There are two strongly interacting peptides representing N-terminal amino acids (51–65 and particularly 91–105) and three peptides representing sequences within the C-terminus of MDM2 (451–465, 461–475 and 481–491). The data also highlight weaker interactions at peptides corresponding to amino acids 151–165 and 171–185 which contains respectively the two established nuclear localisation sequences in MDM2. Weak but detectable interactions were noted at 191–205, 291–305, and 311–325. Strikingly, these data are in excellent agreement with the GST pull-down experiments (Fig 1B). (The five major interacting peptides in the N- and C-terminal regions of MDM2 are shown aligned with the equivalent sequences in MDM4 in S3 Fig. These alignments highlight a number of significant differences in amino acid sequences in at least four of the peptides, especially the peptide corresponding to amino acids 91–105 of MDM2 which showed the greatest degree of interaction (Fig 2A). These differences in sequence may explain why MDM4 could not be co-immunoprecipitated with the anti-MAGE-A antibody (Fig 1).)

The MDM2 C-terminal peptides to which MAGE-A2 binds are located within the RING finger domain which is crucial for homo-dimerization and hetero-dimerization with MDM4. This region also plays a critical role in interacting with, and in mediating the transfer of ubiquitin from, E2 ligases to substrates [39,40,41,42,43]. Moreover, when viewed in the context of the 3D structure of the MDM2 RING, these peptide sequences lie juxtaposed on the surface of the RING that has been proposed to mediate contact with the E2 ligase, UbcH5 ([39,40,42]; Fig 2C). Additionally, they contain several amino acids that have been demonstrated by mutagenesis analysis to play a crucial role in MDM2-dependent p53 ubiquitylation and auto-ubiquitylation [39,40,41,42,43]. The binding of MAGE-A2 to this region suggests that it could influence MDM2-dependent ubiquitylation, possibly in an inhibitory manner.

### MAGE-A2 interacts with the N-terminus of MDM2 *in vitro* in a similar manner to p53

**Fig 3:**
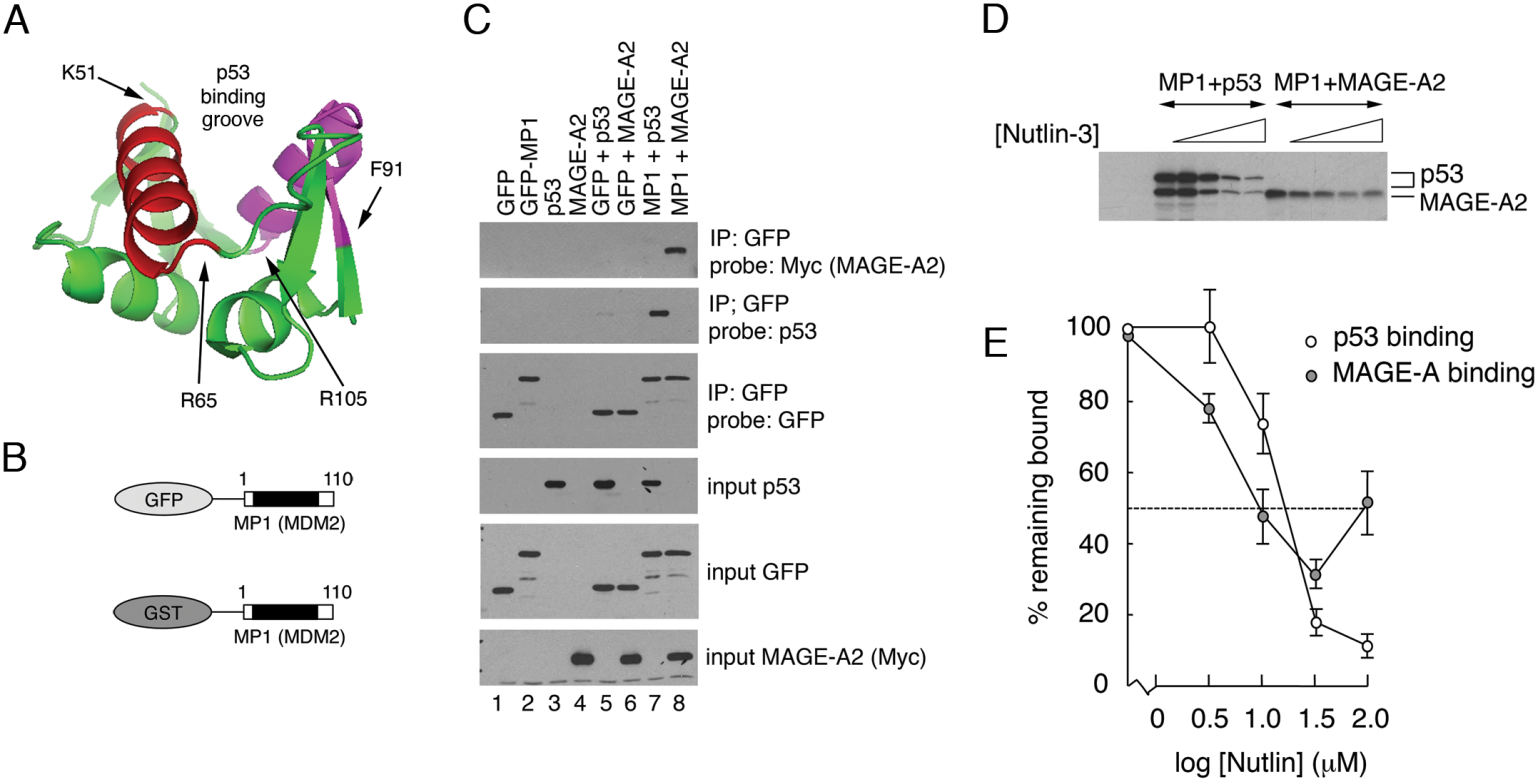
MAGE-A2 interacts with the p53-binding hydrophobic cleft in the N-terminus of MDM2. (A) The structure of the N-terminus (p53-binding domain) of MDM2 (1YCR) showing the positions of MAGE-A2 binding peptides 51–65 (red) and 91–105 (magenta). The backbone is shown in green. The locations of the amino acids at the flanks of each of these peptides are shown. (B) Schematic showing the structure of the GST-MDM2 mini-protein, MP1, used in this experiment. A version in which GST was substituted by GFP and was used for cultured cell expression and immunoprecipitation analysis. (C) Co-immunoprecipitation analysis was carried out following expression of GFP-MP1 (or GFP alone as control) in H1299 cells together with MAGE-A2 or p53 (as positive control). (D) GST pull-down assays were performed in which ^35^S-radiolabelled MAGE-A2, or ^35^S-radiolabelled p53 as control, were captured on glutathione sepharose 4B beads using GST linked to the MDM2 mini-protein, MP1, which encompasses amino acids 1–110 including the hydrophobic p53-binding cleft. The association of MAGE-A2 or p53 was measured in the presence of increasing concentrations of Nutlin-3a. The co-precipitating MAGE-A2 and p53 proteins were detected by SDS-PAGE followed by fluorography. Note that *in vitro* transcription/translation of p53 gives rise to two polypeptides. (E) Quantification of the pull-down experiment following densitometry. In all cases the data are representative of at least three independent experiments.

Further examination of the association of MAGE-A2 with the N-terminus of MDM2 showed that the two interacting peptides represent respectively each side of the hydrophobic grove that is responsible for mediating the interaction with the N-terminal domain of p53. The positions of these peptides within the crystal structure of the MDM2 N-terminal region [44] are highlighted in red and magenta respectively in Fig 3A. Amino acids within both of these regions are required for p53 binding [44] suggesting either that MAGE-A2 may interact with MDM2 in a manner similar to that of p53 or that it may influence the p53/MDM2 interaction. To determine whether MAGE-A2 binds to the N-terminus of MDM2 in a cellular context, the "MP1" MDM2 mini-protein (comprising amino acids 1–110 of MDM2) was expressed as a fusion with green fluorescent protein (GFP; Fig 3B) together with MAGE-A2 in H1299 cells. As a positive control, p53 was expressed in place of GFP-MP1. Co-immunoprecipitation analysis (Fig 3C) confirmed that both MAGE-A2 and the p53 control were able to associate with the GFP-MP1(MDM2) in the cultured cells. No association was observed when GFP lacking the MP1 extension was used.

To test the idea that p53 and MAGE-A2 bind to MDM2 in a similar manner, GST pull-down experiments were carried out *in vitro* to determine whether the competitive inhibitor, Nutlin-3a, which mimics p53 and binds tightly within the hydrophobic groove in MDM2 that constitutes the p53 binding site, could block the interaction of MAGE-A2 with MDM2 [45]. The data show that increasing concentrations of Nutlin-3a do indeed prevent MAGE-A2 from binding to the N-terminus of MDM2 (Fig 3D and 3E). The interaction of p53 with MDM2, which was measured in the same experiment as a control, was also progressively inhibited by increasing concentrations of Nutlin-3a (Fig 3D and 3E). The IC_50_ values for dissociation of p53 and MAGE-A2 respectively from MDM2 were 18 μM and 10 μM indicating that p53 binds to this region comparatively better than MAGE-A2. Curiously, higher levels of Nutlin-3a actually reversed inhibition and stimulated binding of MAGE-A2 to MDM2, an effect that did not occur with p53. One possible explanation of this effect is that prior binding of p53 (or a p53 analogue) may influence the subsequent binding of MAGE-A2.

### MAGE-A2 blocks MDM2-mediated ubiquitylation in cultured cells but does not affect p53 turnover

**Fig 4:**
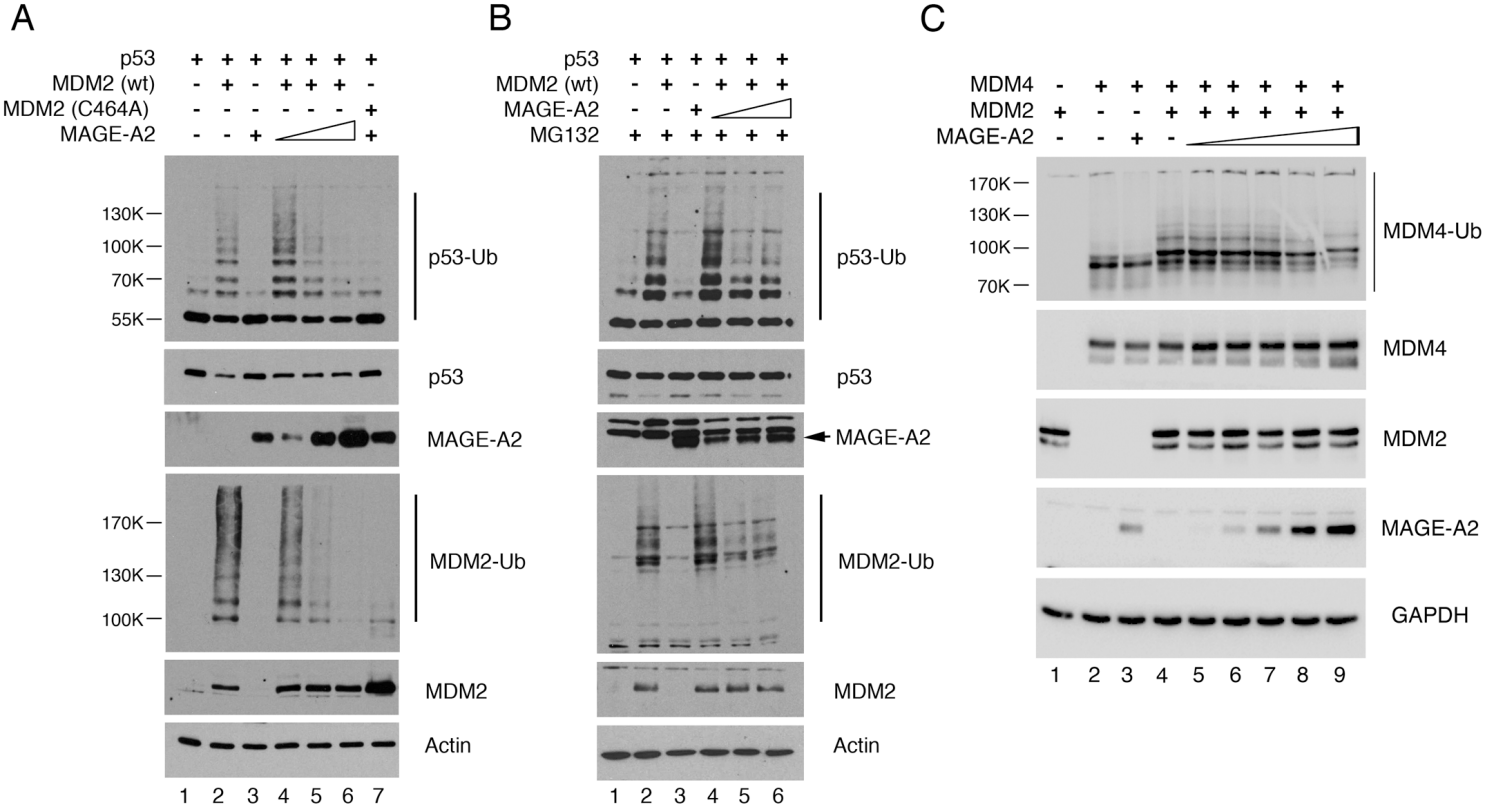
MAGE-A2 is a regulator of MDM2 ubiqutylation function. (A) H1299 cells were seeded on 10 cm dishes at a density of 10^6^ cells per plate and transfected with a plasmid expressing His-tagged ubiquitin together with plasmids expressing p53, wild type MDM2, a C464A mutant of MDM2 and increasing amounts of a plasmid expressing HA-tagged MAGE-A2 (0.5, 1.0 and 2.0 μg respectively) as indicated. Following lysis, the proteins were detected by western blotting or, in the case of the ubiquitylated proteins, were first captured on Ni^2+^-agarose beads followed by detection by western blotting. (B) The experiment described in panel A was carried out following treatment of the cells with 10 μM MG132 prior to cell lysis. (C) An experiment similar to that described in panel A was carried out in which p53 was substituted as the substrate for ubiquitylation by MDM2. The amounts of MAGE-A2 expressing plasmid were: 0.25, 0.5, 1.0, 1.5 and 2.0 μg. In each panel, the data are representative of at least three independent experiments.

To determine whether indeed MAGE-A proteins can act as MDM2 regulators, an established cell culture-based ubiquitylation assay was used [35] in which H1299 cells were co-transfected with plasmids expressing wild-type human p53, MDM2, His-tagged ubiquitin, and increasing amounts of MAGE-A2. At 36 h post-transfection, the cells were lysed in 6M guanidinium buffer, conditions that prevent deubiquitylation of proteins and disrupt any non-covalent protein—protein interactions. The His-tagged ubiquitylated proteins were purified by affinity chromatography using Ni^2+^-agarose beads, then subsequently eluted and analyzed by Western blotting with the anti-p53 antibody, DO-1. The data (Fig 4A, upper panel) confirmed that MDM2 was able to ubiquitylate p53 in this assay and, importantly, decrease p53 levels. Notably, p53 was not ubiquitylated by MAGE-A2 alone (compare lanes 1, 2 and 3). When used in combination, low levels of MAGE-A2 gave at best a marginal increase in p53 ubiquitylation but, at higher levels, led to a significant dose-dependent decrease in the levels of p53 ubiquitylation mediated by MDM2 (lanes 4–6). Strikingly, however, although these changes in MDM2 ubiquitylation function were clearly evident, there was no corresponding effect on p53 turnover (second panel from top). A ubiquitylation-defective mutant of MDM2 (C464A, lane 7) failed to either ubiquitylate or degrade p53 and confirmed that the ubiquitylation of p53 in this experiment was mediated principally by MDM2 and not by an endogenous E3 ligase. When the auto-ubiquitylation function of MDM2 was examined, a significant decrease in MDM2 autoubiquitylation was observed as MAGE-A2 levels were increased. As with p53, the levels of MDM2 were unaffected (bottom panel). Additionally, the turnover of MDM2, as measured by cycloheximide-chase, was unaltered (S4 Fig).

To confirm that the changes in p53 ubiquitylation were dependent on the ubiquitylation function of MDM2 *per se*, and did not simply reflect any alterations in p53 proteins levels, the experiment was carried out in the presence of the proteasome inhibitor, MG132, to block protein degradation. The data (Fig 4B) confirmed that changes in p53 levels do not account for the observed inhibition of p53 ubiquitylation by MAGE-A2. Another possible explanation for the reduced levels of ubiquitylation is that MAGE-A2 itself is a substrate for MDM2 and can therefore act as a competitive inhibitor of other substrates under our assay conditions. However, we were unable to detect any ubiquitylation of MAGE-A2 by MDM2 under conditions where MDM2 is able to ubiquitylate p53 and MDM2 itself, thereby ruling out this possibility.

In addition to regulating p53 levels, MDM2 interacts with, and mediates the ubiquitylation of, many other cellular proteins including MDM4. To determine whether MAGE-A2 could affect the ubiquitylation of MDM4 by MDM2, MDM4 was introduced into the ubiquitylation assay. The data (Fig 4C) indicate that, as with p53 and MDM2, increasing levels of MAGE-A2 led to a detectable decrease in the ubiquitylation of MDM4 by MDM2. (The reduction in MDM4 ubiquitylation, while evident, appears less robust than with the p53 or MDM2 [panels A and B]. We cannot, therefore, rule out the possibility that other cellular E3 ligases or de-ubiquitylases may have an impact on the magnitude of this response.) Strikingly, however, the presence of even low levels of MAGE-A2 led to a small but significant elevation of MDM4 protein levels. These data suggest that, although MAGE-A2 can inhibit the general ubiquitylation function of MDM2, there is a selective outcome in terms of promoting degradation.

### MAGE-A governs the levels of endogenous MDM4

We and others have previously shown that MAGE-A proteins can directly regulate p53 transcription function but show little influence on regulating wild type p53 protein levels [7,12,13]. Contrary to these observations, other researchers have found that MAGE proteins can influence p53 levels independently of MDM2 by regulating the activity of other p53-targeted ubiquitin ligases [8,17]. Upon re-examination, we confirmed that silencing of MAGE-A expression in U2OS cells, in which p53 levels are governed mainly through the action of MDM2, leads to increased levels of the p53-transcriptional target, p21, yet shows at best only a marginal effect p53 protein levels (S5 Fig). These data support the idea that MAGE-A proteins can acutely down-regulate p53 function without necessarily affecting p53 levels and are consistent with the unaltered p53 levels seen in the ubiquitylation experiment (Fig 4).

**Fig 5:**
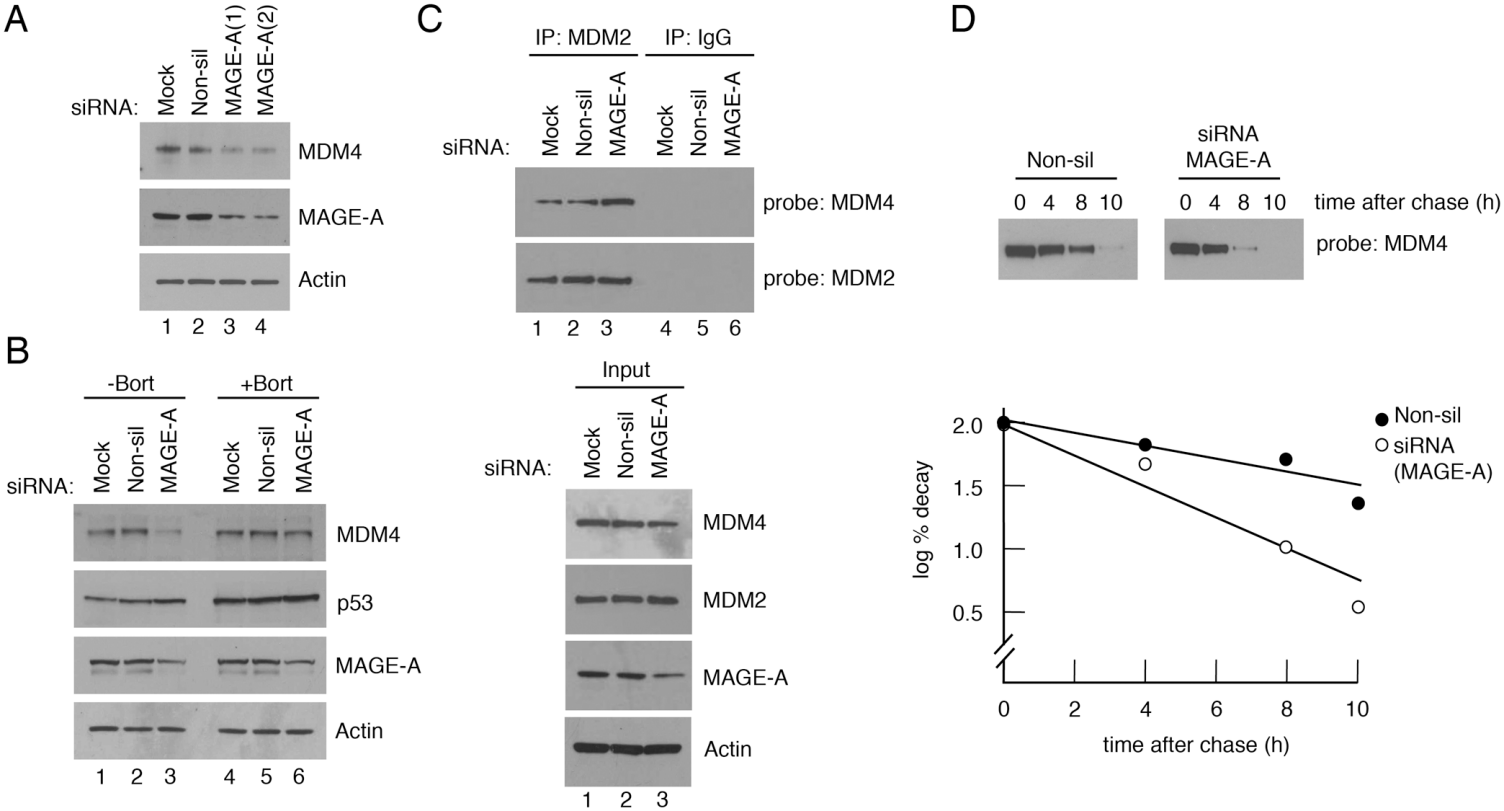
Silencing of MAGE-A expression decreases the levels of MDM4 via the proteasome and increase MDM2/MDM4 association. MAGE-A expression in U2OS cells was silenced by RNAi as previously described or treated with a non-silencing siRNA oligonucleotide as control [12]. The various proteins were detected by western blotting. (A) Silencing of MAGE-A in U2OS cells. (B) Silencing of MAGE-A was carried out in the absence or presence of the proteasomal inhibitor, bortezomid. The cells were treated with 10 μM bortezomib 6 h prior to harvesting. (C) Co-immunoprecipitation analysis in which MDM2 immunoprecipitates (obtaining using the antibody, 4B2) were probed for MDM4 (8C6) and for MDM2 itself. (D) Cycloheximide-chase analysis of MDM4 in the U2OS cells was carried out following treatment with the non-silencing and MAGE-A-silencing siRNA oligonucleotides. Quantification of the western blots was carried out using ImageJ software. In all cases the data shown are representative of at least three independent experiments.

In contrast to the unaltered p53 levels, the data in Fig 4 indicate that elevated expression of MAGE-A2 leads to increased levels of MDM4. To determine whether the ability of MAGE-A to elevate MDM4 observed in the ubiquitylation assay holds true for endogenous proteins, MDM4 levels were examined following silencing of MAGE-A expression in U2OS cells. The data (Fig 5A) show that decreasing the levels of MAGE-A leads to a significant reduction in MDM4 levels. Consistent with a mechanism involving the ubiquitylation and turnover of MDM4, the reduction on MDM4 levels could be partially rescued by the presence of the proteasomal inhibitor, bortezomid (Fig 5B); (bortezomid, like MG132, is a potent inhibitor of protein degradation by the proteasome). To confirm that MAGE-A depletion led to increased turnover of MDM4, cycloheximide-chase analysis of MDM4 was conducted following treatment of the U2OS cells with the non-silencing and MAGE-A-silencing siRNA oligonucleotides. The data indicated that there was a reduction in MDM4 half-life from approximately 6.5 h to 2.5 h as a result of MAGE-A silencing (Fig 5D). Taken together, these data suggest that MAGE-A proteins are inhibitors of MDM2 ubiquitylation function that lead to reduced targeting of MDM4 for degradation.

### Reduced MAGE-A levels lead to increased MDM2/MDM4 association

The representation of the MAGE-A-interacting region at the C-terminus of MDM2 (Fig 2C) suggests that MAGE-A could block the interaction of MDM2 with an important stimulatory or functional partner(s), such as MDM4 itself and/or the E2 ubiquitin ligase UbcH5, both of which interact with this region of MDM2. Given that the interaction of MDM2 with the E2 ligase has been reported by different groups to be weak [40,42], we focused on the possibility that MAGE-A might influence MDM2/MDM4 association. To test this possibility, MDM2/MDM4 association was measured in a co-immunoprecipitation assay following silencing, or mock silencing, of endogenous MAGE-A in U2OS cells. The data (Fig 5C) show that reduction in the levels of endogenous MAGE-A proteins leads to an increase in the amount of MDM4 that is associated with MDM2. These data are consistent with the idea that, mechanistically, MAGE-A proteins can interrupt association of MDM2 with a partner that is required to mediate full ubiquitylation function.

### Detection of MAGE-A antigens in human breast cancers is associated with elevated MDM4 levels

The molecular analyses described above predict that cancers showing detectable MAGE-A expression should also show elevated levels of MDM4. To determine whether this relationship holds true in human cancers immunohistochemical (IHC) analysis of MAGE-A and MDM4 expression in a well-characterized cohort of 225 primary human breast cancers was carried out. Analysis of the data (Table 1) indicated that MAGE-A was detected in a small proportion of breast cancers (2%). Nevertheless there was a significant association between MAGE-A expression and elevated MDM4 such that there is a greater than 6-fold likelihood of having increased intensity of MDM4 staining if the tumour shows the presence of MAGE-A; (elevated MDM4 levels were judged on the basis of low versus medium/high staining). While MAGE-A expression is likely to be only one mechanism by which MDM4 levels can be increased, these data support the idea that re-expression of MAGE-A can indeed lead to increased MDM4 levels during the development of human disease.

**Table 1:** Immunohistochemical analysis of MAGE-A and MDM4 in a cohort of 225 human primary breast cancer specimens.

		MAGE-A	staining		^a^P value	^b^Odds Ratio
		Neg [N(%)]	Pos [N(%)]	Total [N(%)]		
**MDM4 staining**	low	187 (83.1)	4 (1.8)	191 (84.9)	0.0197	6.2
	high	30 (13.3)	4 (1.8)	34 (15.1)		
	total	217 (96.4)	8 (3.6)	225 (100)		

^a^The P value was achieved using Fisher's exact test

^b^The Odds ratio determines the likelihood of elevated MDM4 staining if the tumor is MAGE-A positive as opposed to MAGE-A negative.

## Discussion

MAGE-A comprises an 11-member sub-family of the broader family of MAGE proteins which are characterised by the presence of a MAGE-homology domain [3]. MAGE-A proteins (and other MAGE family members) can influence the function of certain transcription factors and act as regulatory partners for various RING finger-type ubiquitin E3 ligases [17,46]. Current evidence suggests that there is a significant degree of functional overlap between many MAGE-A sub-family members, at least at the biochemical level. These functions may contribute to the ability of the MAGE-A proteins to actively promote malignancy.

In the present study, we find a direct biochemical and cellular interaction between MAGE-A proteins and the RING finger-type ubiquitin E3 ligase, MDM2. We identify specific domains of MDM2 that interact directly with MAGE-A2 including, principally, the N-terminal hydrophobic pocket that normally serves as a crucial binding site for p53, and the E2-binding surface within the C-terminal RING structure. Consistent with interaction through these points of contact we show that MAGE-A2 can act as a potent inhibitor of MDM2 ubiquitylation function towards p53 and its regulatory partners, MDM4 and MDM2 itself. This is the first demonstration, to our knowledge, that MAGE proteins can act as inhibitors as opposed to activators of RING finger ligases. Additionally, we have shown previously that basal (unstimulated) levels of MDM2 are suppressed by MAGE-A through its ability to inhibit p53 function [12]. Our data suggest, therefore, that MAGE-A can reduce MDM2 function both directly, through inhibiting its ability to ubiquitylate substrates, and indirectly by down-regulating its transcription.

**Fig 6:**
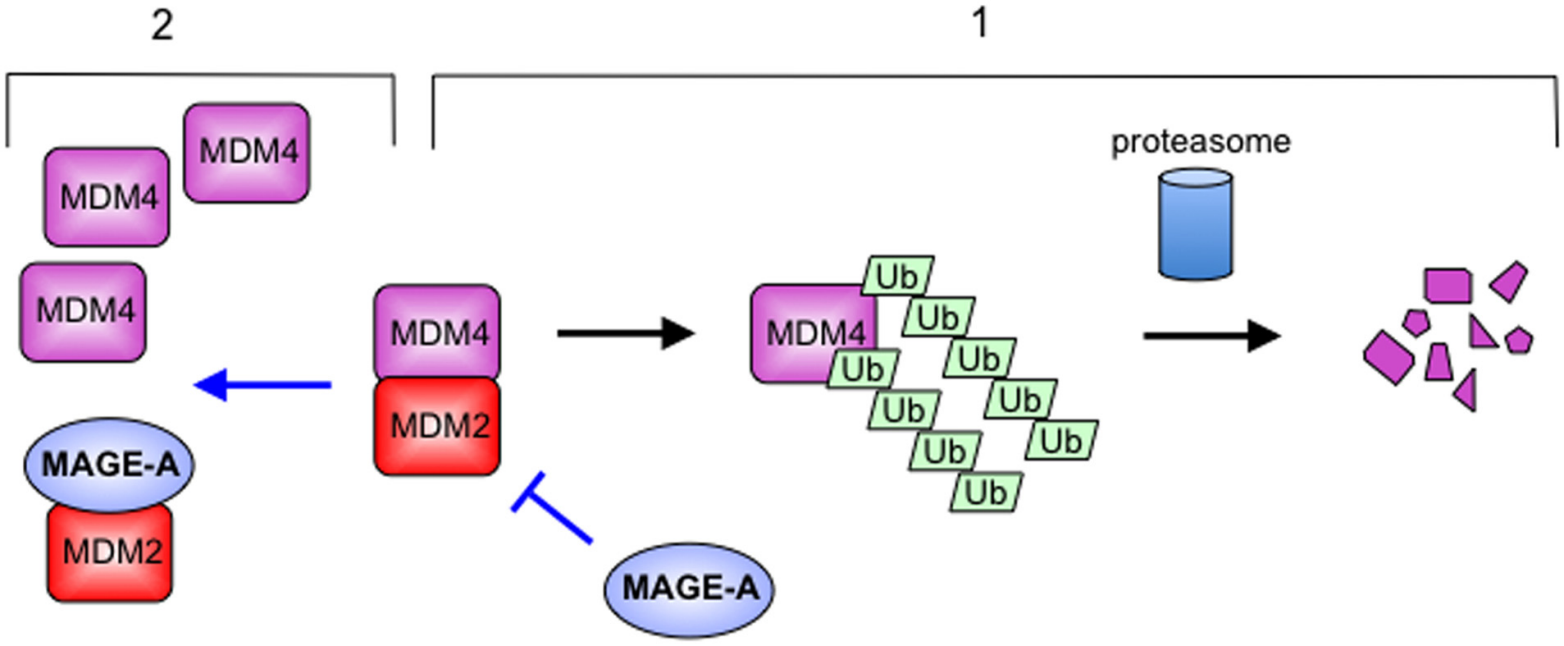
Model depicting the effect of MAGE-A on MDM2 and MDM4. MDM2 and MDM4 interact through their respective RING finger domains (located at their C-termini). This interaction allows both the activation of MDM2 ubiquitylation function and the ubiquitylation of MDM4 itself leading to MDM4 destruction. MAGE-A interacts preferentially with MDM2 via the N-terminal hydrophobic pocket and the RING finger. The model predicts that this will compete with MDM4 for binding to MDM2, leading to elevated levels of MDM4.

A model describing the behaviour of MAGE-A towards MDM2 and the outcome of their interaction is given in Fig 6 and can be summarised as follows. In addition to ubiquitylating p53, MDM2 can ubiquitylate MDM4 (and auto-ubiquitylate) leading to their degradation (Fig 6, right hand side). (The balance between p53 ubiquitylation and MDM2/MDM4 ubiquitylation is normally regulated by signals that impact on the p53 pathway and is summarised elsewhere [24,25].) When MAGE-A proteins are present they are able to interact with MDM2, both at its N-terminus and through an extensive region within the RING finger domain (that encompasses interaction sites for both the E2 ligase and MDM4), but they do not bind detectably to MDM4 itself, at least in our co-immunoprecipitation analysis. The data in Fig 6C suggest that the binding of MAGE-A to the MDM2 RING can disrupt MDM2/MDM4 association (left hand side). MAGE-A binding may also interfere with the interaction between the MDM2 RING and the E2 ligase, or it is possible the impairment of this interaction is a consequence of disrupting MDM2/MDM4 contact [41]. This would explain why increased levels of MAGE-A lead to a significant decrease in the ubiquitylation of the three substrates tested (p53, MDM2, MDM4: Fig 4). The additional contact of MAGE-A with the N-terminal part of MDM2 responsible for binding p53 would underpin this inhibition. The outcome for MDM4 is that it is uncoupled from MDM2 leading to an increase in its levels.

The model does not explain why inhibition of p53 ubiquitylation and MDM2 auto-ubiquitylation fails to interrupt p53 and MDM2 turnover (Fig 4, S4 and S5 Figs). There are several possible explanations for this result: (i) it is possible that, under our experimental conditions, a low level of p53 ubiquitylation remains, even in the presence of high levels of MAGE-A expression, that is sufficient to mediate efficient p53 turnover by the proteasome. (ii) Given that the ubiquitylation and degradation functions of MDM2 are separable experimentally and are regulated independently; (e.g. phosphorylation of the MDM2 acidic domain regulates MDM2 mediated turnover but does not affect ubiquitylation [22]), the binding of MDM2 to MAGE-A may be able to "substitute" for ubiquitin and permit targeting of p53 and MDM2 to the proteasome. (iii) Given that p53 can be ubiquitylated by as many as 15 different E3 ligases [47], and that MAGE-A has been shown to promote p53 turnover through stimulating the function of at least one of these (TRIM28/KAP1: [8,17]), MAGE-A may redirect the ubiquitylation of p53 through a different route but retain that function of MDM2 that is required for proteasomal targeting and/or degradation. Future studies should hopefully resolve these issues.

The major outcome of the model in Fig 6 is that MAGE-A will cause the cellular levels of MDM4 to rise. MDM4 works as a stimulatory partner for MDM2, at least in cultured cells, although this function may be restricted to certain stages of development and therefore less important in adults [48,49]. MDM4 also acts as a potent inhibitor of p53 transactivation function by binding to the transcriptional activation domain of p53 and sterically blocking its interaction with the transcriptional machinery [30]. A MAGE-A-dependent increase in intracellular MDM4 is therefore likely to lead to, or contribute to, p53-mediated transcription being reduced. Alternatively, given that MDM4 may selectively affect the expression of some p53-responsive genes but not others [50,51], elevated MDM4 could impact on the p53 transcriptional programme. We and others have previously established that MAGE-A suppresses p53 transcriptional function through mechanisms including the targeting of histone deacetylase activity to p53, interfering with PLMIV-promoted p53 activation, and sterically blocking p53 from interacting with target sites in responsive promoters [12,13,16]. The finding that MAGE-A increases MDM4 levels in addition to these other mechanisms suggests that MAGE-A mediates a coordinated inhibition of p53 function through controlling various complementary biochemical functions.

Elevated levels of MDM4 in cancers that express wild type p53 is a common event that is regarded as a mechanism for p53 inactivation during tumorigenesis [25]. There is also evidence that MDM4 can contribute to cancer through p53- and MDM2-*independent* mechanisms leading to increased genomic instability [52]. In these contexts the observed association between MAGE-A expression and increased MDM4 levels in primary breast cancer is quite striking and suggestive of a mechanistic link between these proteins during cancer development. Given, however, the small proportion of the breast cancers that express detectable MAGE-A protein, this association should be explored in greater depth in cancers where MAGE-A and MDM4 increases are more common. In malignant melanomas, for example, where p53 mutation is unusual, elevated MDM4 levels have been reported in >65% of cases [53]. Curiously, melanomas also frequently express high levels of MAGE-A (in approximately 50% of cases) and other Cancer/Testis antigens [54]. It will be interesting therefore to interrogate cohorts of these and other cancer types to challenge not only the link between MAGE-A expression and MDM4 levels but also their relationship to p53 wild type status. Robust analysis of this type could significantly underpin the relevance of the above model to human disease.

## Supporting Information

S1 Fig H1299 and U2OS cells express members of the MAGE-A family. The expression of MAGE-A was determined: (A) by RT-PCR using primers specific for individual family members and carried out as described previously [12]; and (B) by western blotting using the pan-MAGE-A antibody, 6C1.
(TIF)

S2 Fig MDM2 representative peptides used in the pepscan assay. Biotinylated peptides were anchored to streptavidin-coated beads and use to captured ^35^S-labelled MAGE-A2 as described in the Materials and Methods section. The peptides are 15 amino acids in length and overlap with the next peptide in sequence by 5 amino acids. The MDM2 sequence is given above and the peptides are numbered consequently and are represented as black lines underneath the appropriate amino acid sequence. The peptides that bind tightly to MAGE-A2 are highlighted in yellow while those that bind weakly are highlighted in grey (containing the nuclear localization sequences) or lilac (containing the nuclear export sequence).
(TIF)

S3 Fig MAGE-A2 associates with the C-terminus of MDM2 at the RING finger and interacts with the surface that mediates contact with E2 ubiqutin ligases. The MDM2-representing peptides that bind tightly to MAGE-A are shown in black and the amino acid positions are numbered. The corresponding sequences in MDM4 are shown below these in blue. Amino acid identities are represented by asterisks while conservative changes are indicated by dots.
(TIF)

S4 Fig MDM2 turnover is not affected by MAGE-A co-expression. H1299 cells were transfected with plasmids expressing MDM2 together with MAGE-A2 or empty vector. 36 h post-transfection, the cells were treated with cycloheximide (CHX; 10 μg/ml) and harvested at the indicated time points. (A) Extracts were analysed by western blotting using the SMP-14 and 4B2 anti-MDM2 antibodies. (B) The signals obtained for MDM2 were quantitated by densitometry.
(TIF)

S5 Fig p53 levels are not significant affected by following silencing of MAGE-A in U2OS cells. Expression of MAGE-A was silenced in U2OS cells using two independent siRNA oligonucleotides as described in Materials and Methods. The various proteins were detected by western blotting.
(TIF)

S6 Fig Uncropped gels from data presented in the figures. (PDF)
