# Supplementary figures and images for: MAGE-A Cancer/Testis Antigens Inhibit MDM2 Ubiquitylation Function and Promote Increased Levels of MDM4
Source: PLoS One. 2015 May 22;10(5):e0127713. doi: 10.1371/journal.pone.0127713 (PMC4441487; doi:10.1371/journal.pone.0127713)

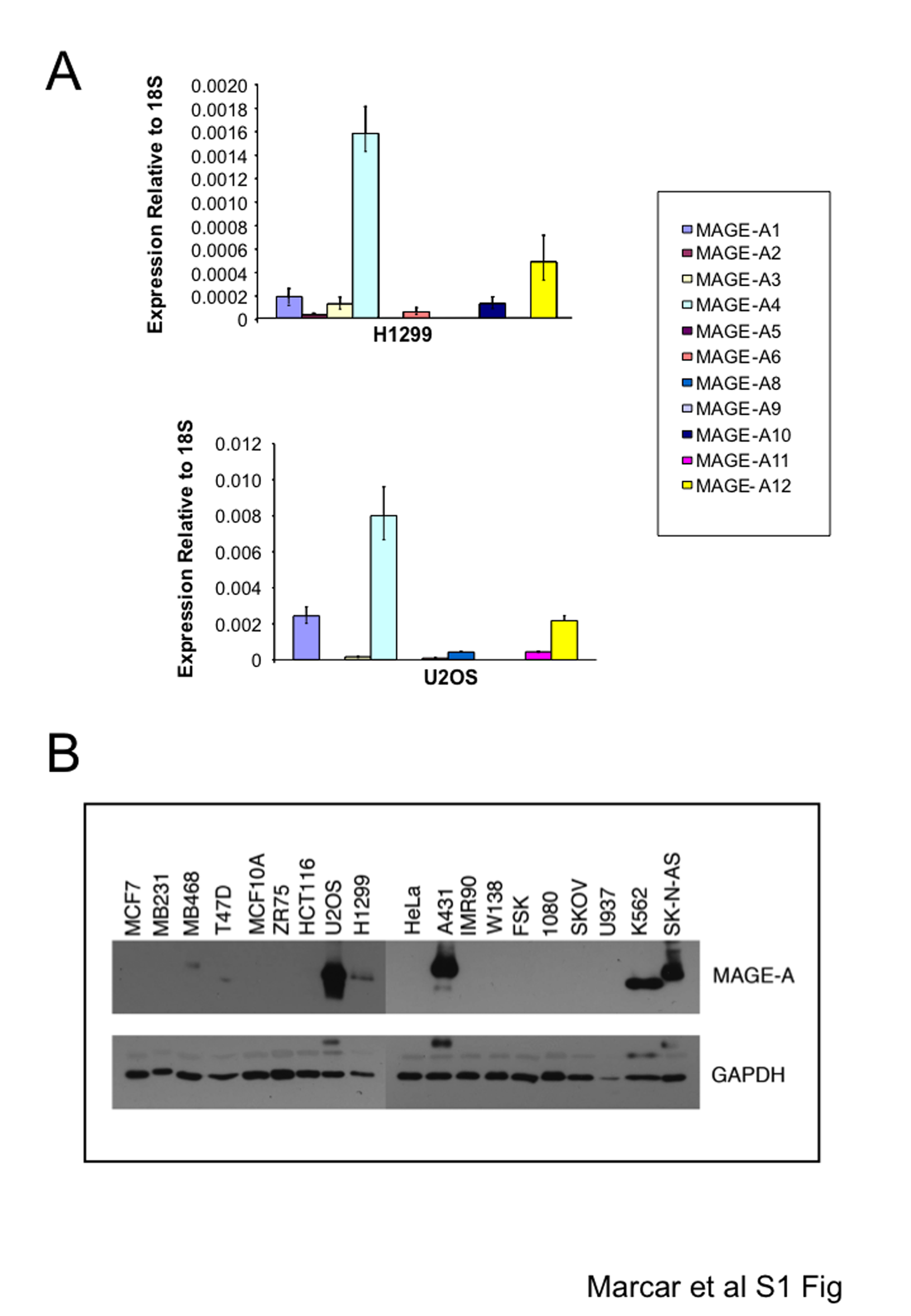

Supplement: S1 Fig — The expression of MAGE-A was determined: (A) by RT-PCR using primers specific for individual family members and carried out as described previously [12]; and (B) by western blotting using the pan-MAGE-A antibody, 6C1. (TIF) [file pone.0127713.s001.tif]

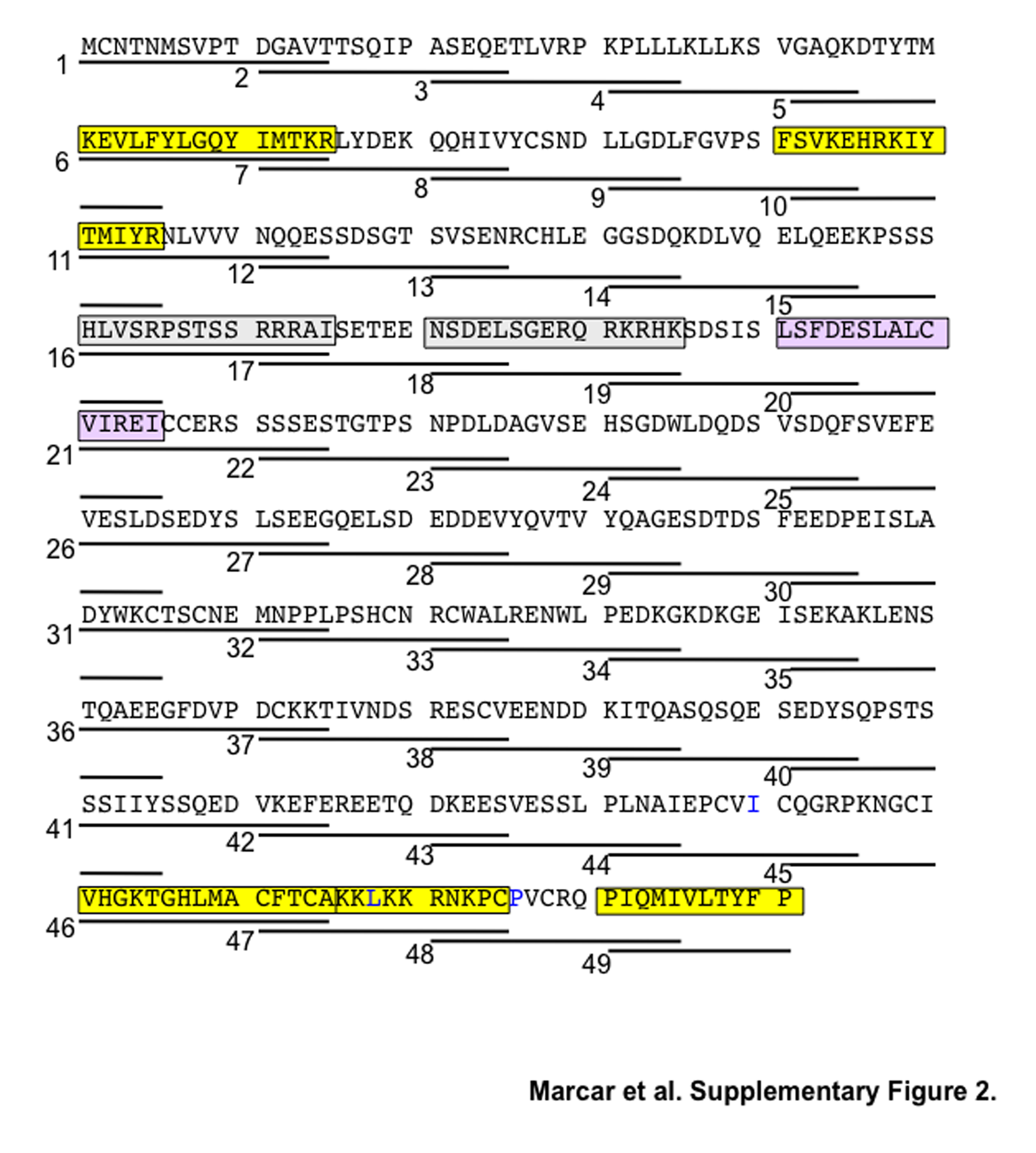

Supplement: S2 Fig — Biotinylated peptides were anchored to streptavidin-coated beads and use to captured 35S-labelled MAGE-A2 as described in the Materials and Methods section. The peptides are 15 amino acids in length and overlap with the next peptide in sequence by 5 amino acids. The MDM2 sequence is given above and the peptides are numbered consequently and are represented as black lines underneath the appropriate amino acid sequence. The peptides that bind tightly to MAGE-A2 are highlighted in yellow while those that bind weakly are highlighted in grey (containing the nuclear localization sequences) or lilac (containing the nuclear export sequence). (TIF) [file pone.0127713.s002.tif]

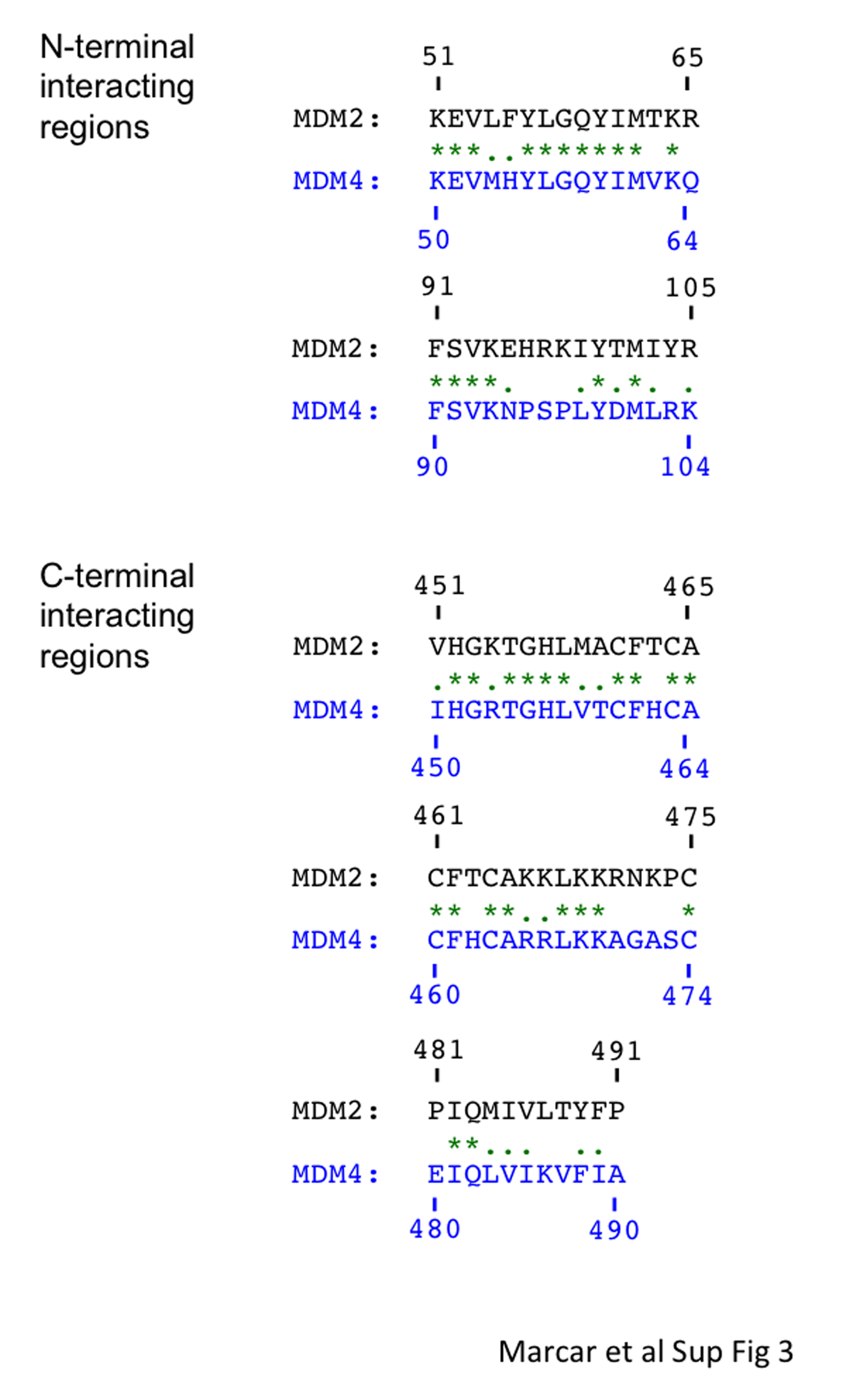

Supplement: S3 Fig — The MDM2-representing peptides that bind tightly to MAGE-A are shown in black and the amino acid positions are numbered. The corresponding sequences in MDM4 are shown below these in blue. Amino acid identities are represented by asterisks while conservative changes are indicated by dots. (TIF) [file pone.0127713.s003.tif]

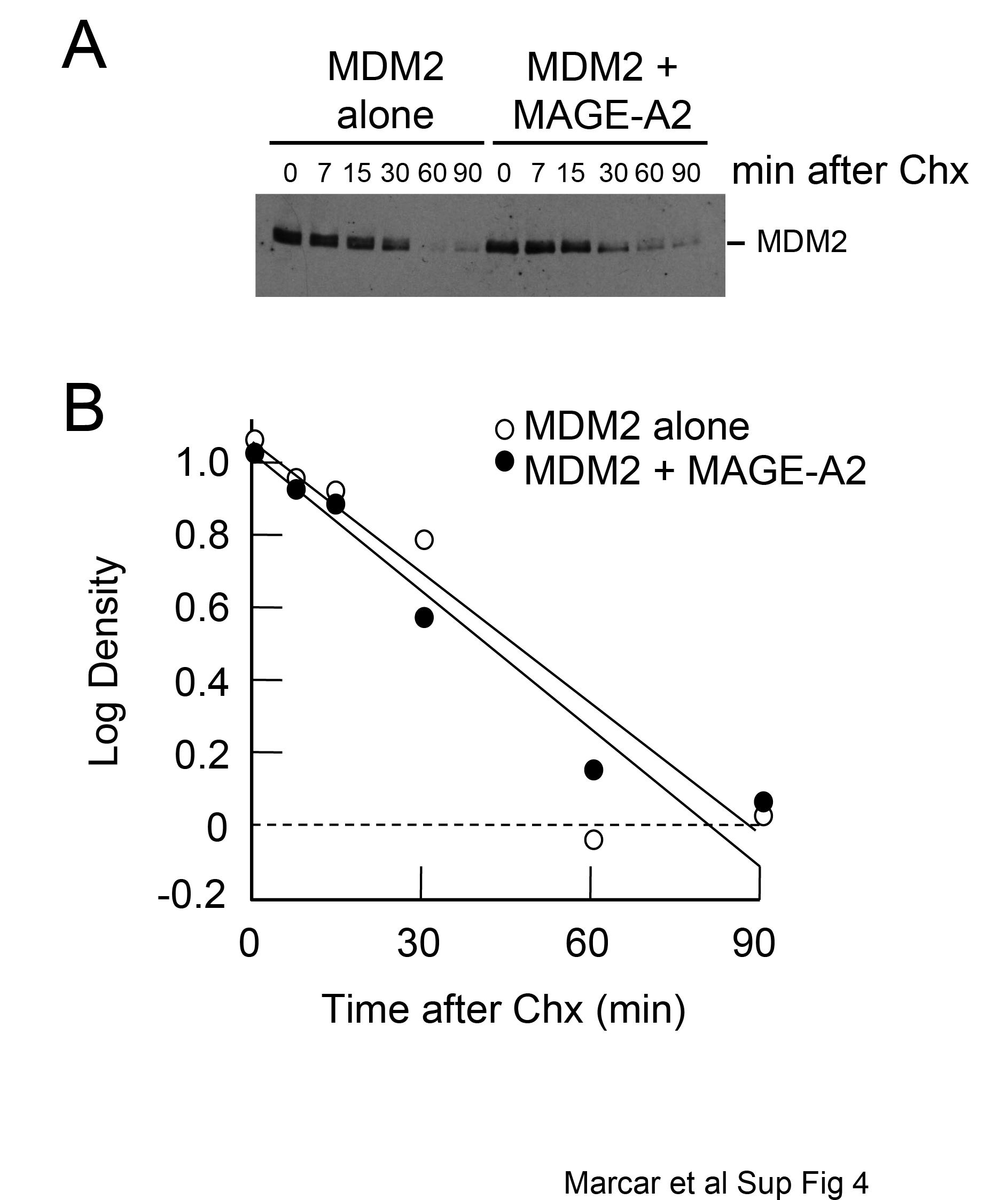

Supplement: S4 Fig — H1299 cells were transfected with plasmids expressing MDM2 together with MAGE-A2 or empty vector. 36 h post-transfection, the cells were treated with cycloheximide (CHX; 10 μg/ml) and harvested at the indicated time points. (A) Extracts were analysed by western blotting using the SMP-14 and 4B2 anti-MDM2 antibodies. (B) The signals obtained for MDM2 were quantitated by densitometry. (TIF) [file pone.0127713.s004.tif]

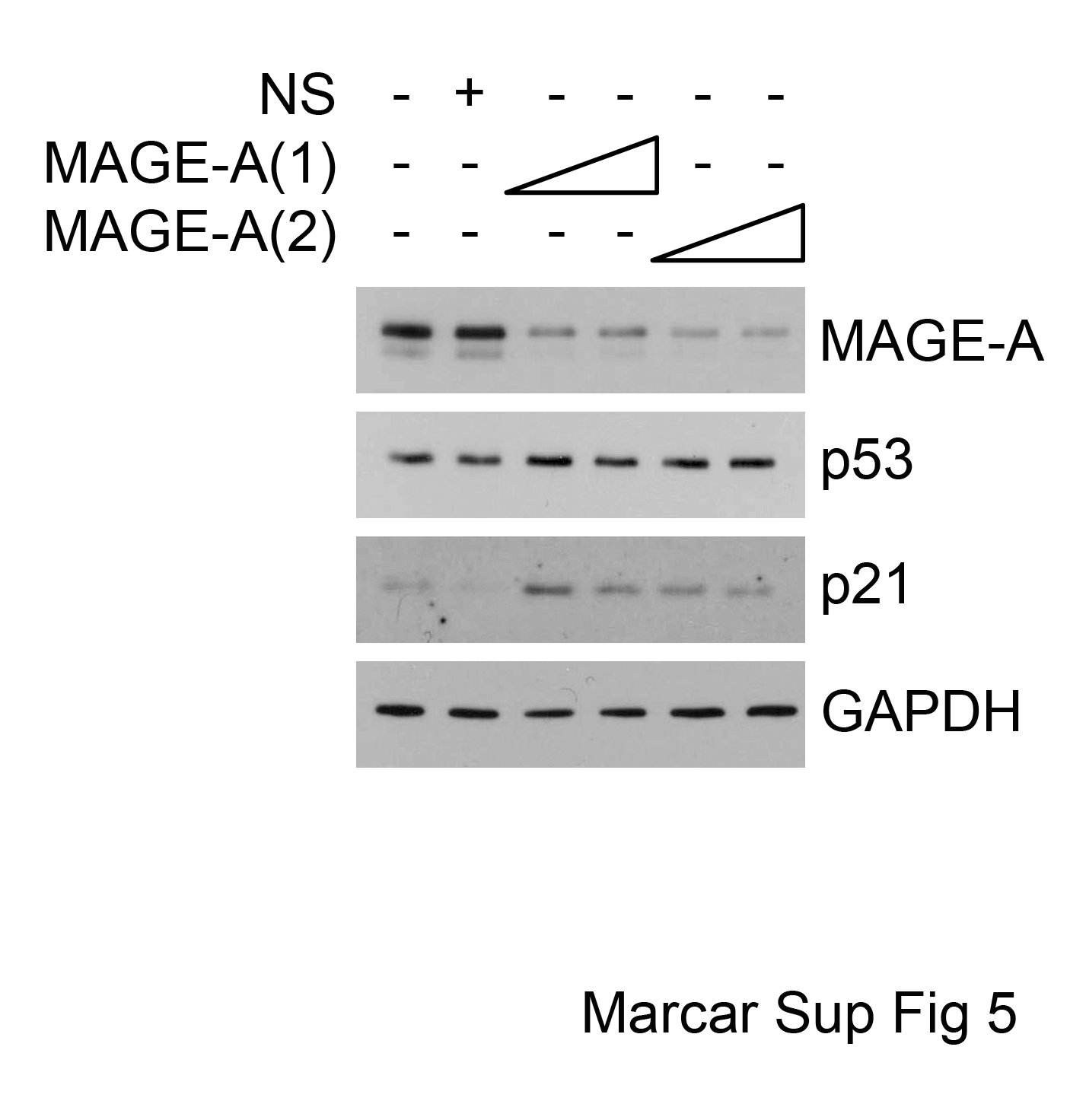

Supplement: S5 Fig — Expression of MAGE-A was silenced in U2OS cells using two independent siRNA oligonucleotides as described in Materials and Methods. The various proteins were detected by western blotting. (TIF) [file pone.0127713.s005.tif]
