# Supplementary material for: MAGE-A Cancer/Testis Antigens Inhibit MDM2 Ubiquitylation Function and Promote Increased Levels of MDM4
Source: PLoS One. 2015 May 22;10(5):e0127713. doi: 10.1371/journal.pone.0127713 (PMC4441487; doi:10.1371/journal.pone.0127713)
Supplement: S6 Fig — (PDF) [file pone.0127713.s006.pdf]

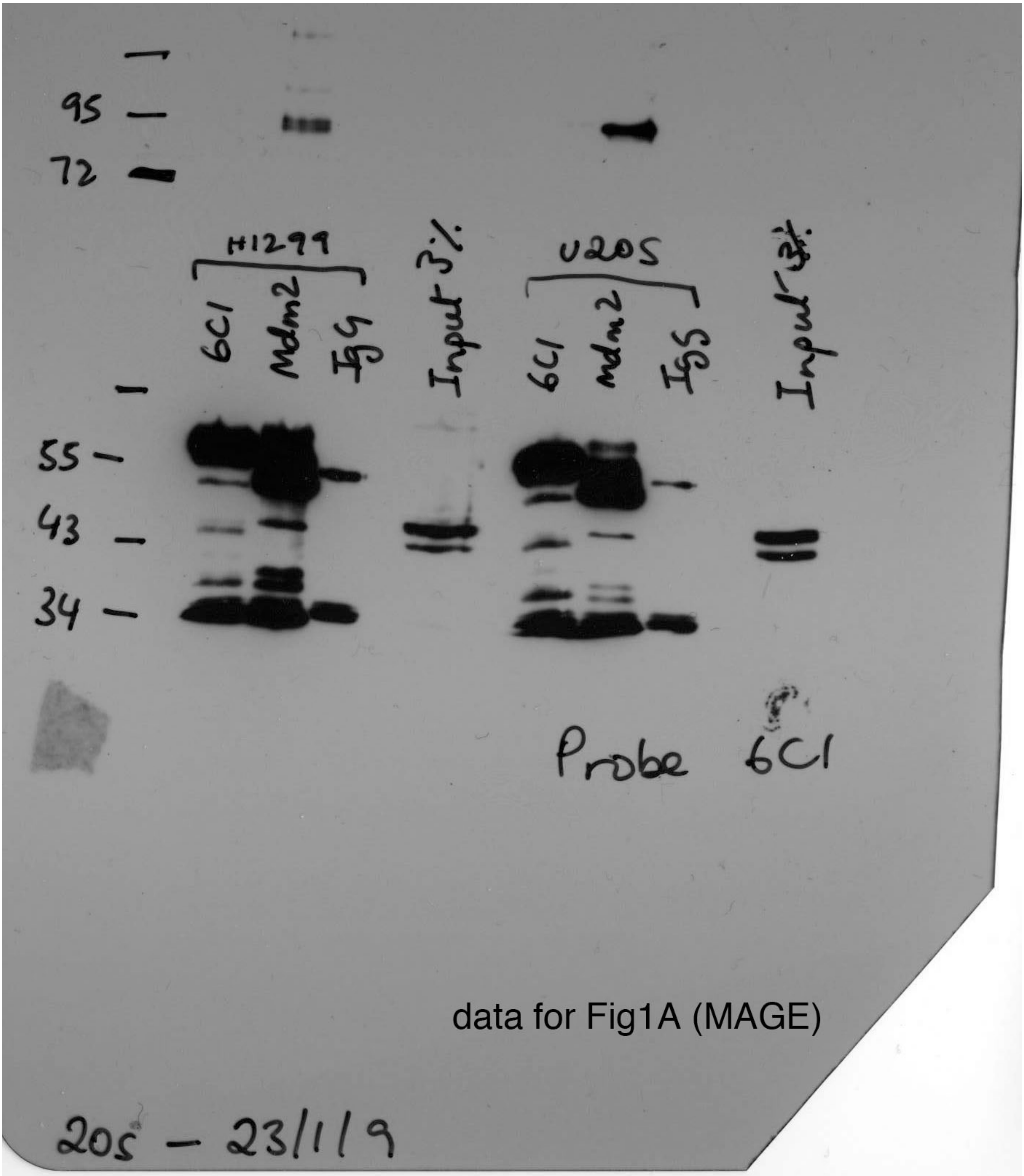

data for Fig1A (MAGE)

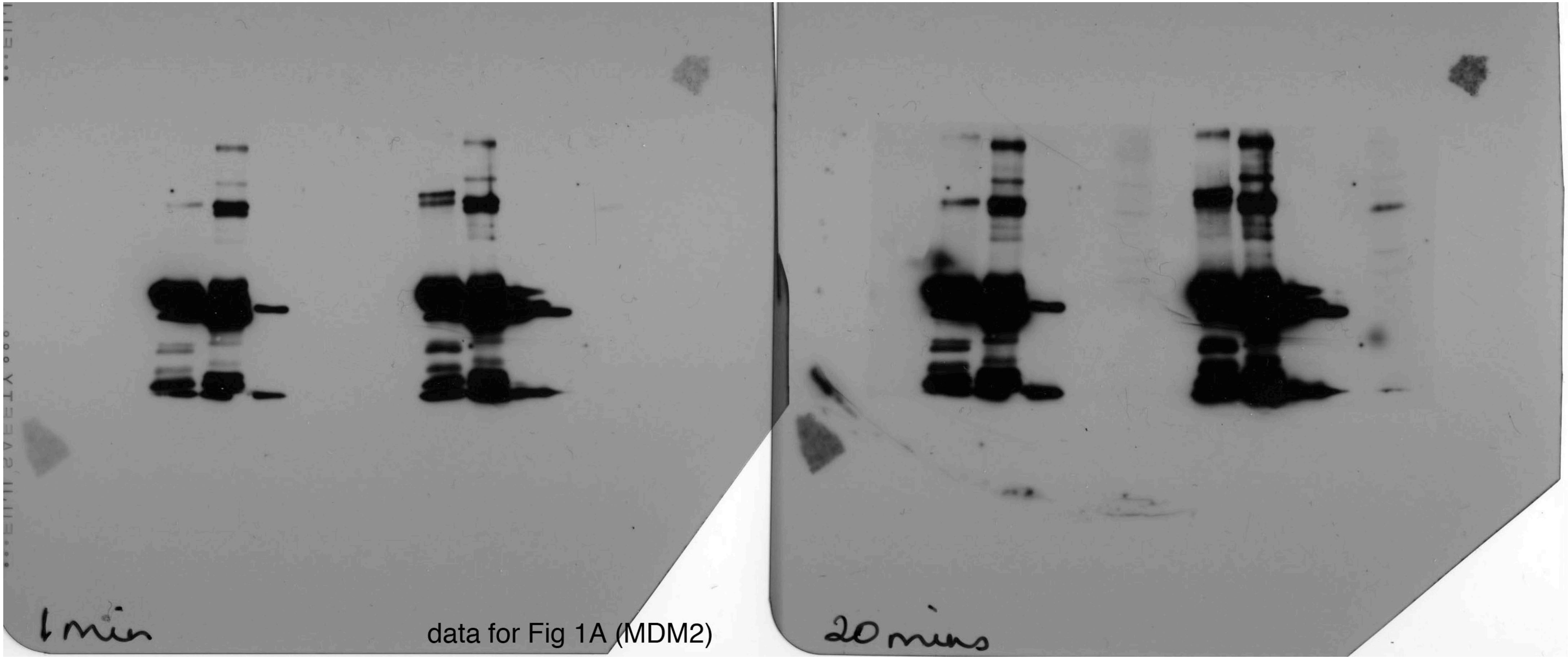

data for Fig 1A (MDM2)

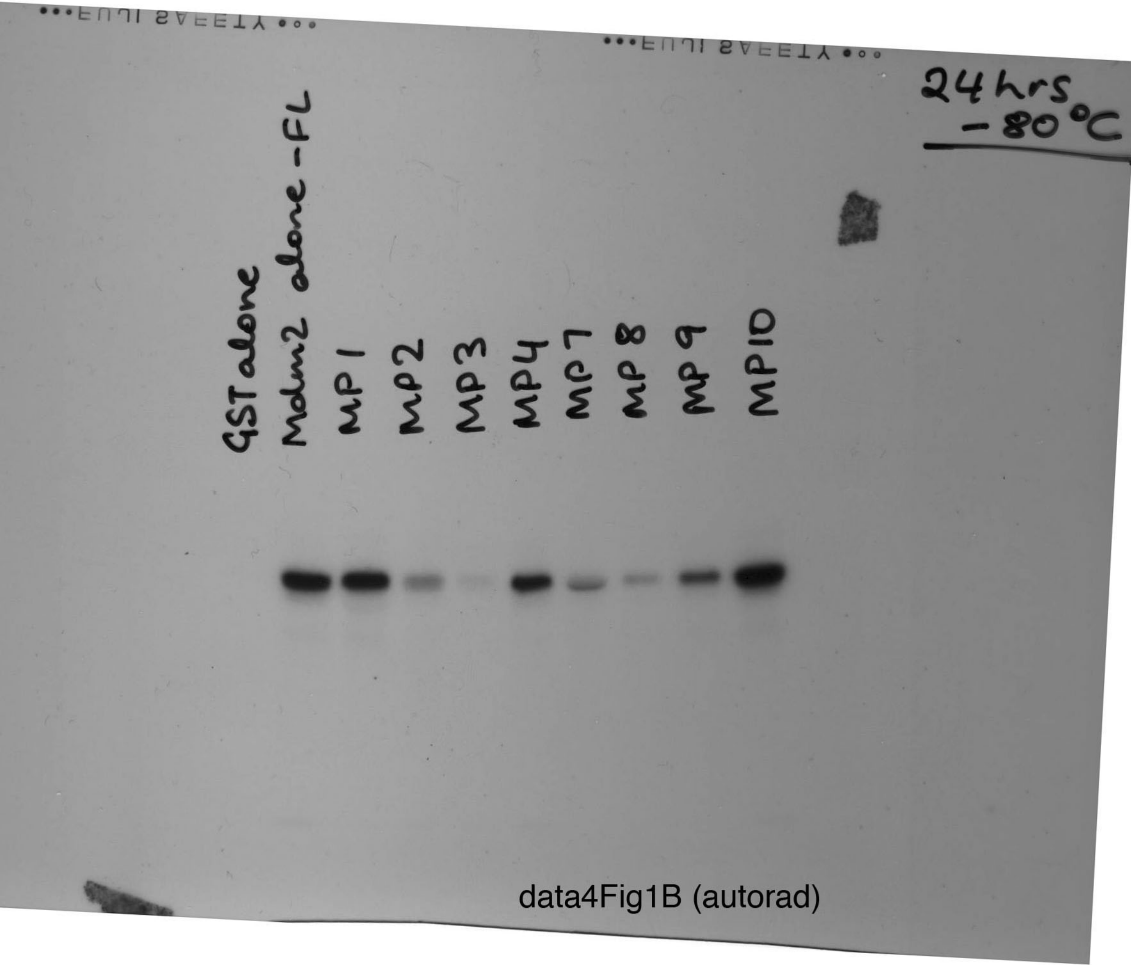

data4Fig1B (autorad)

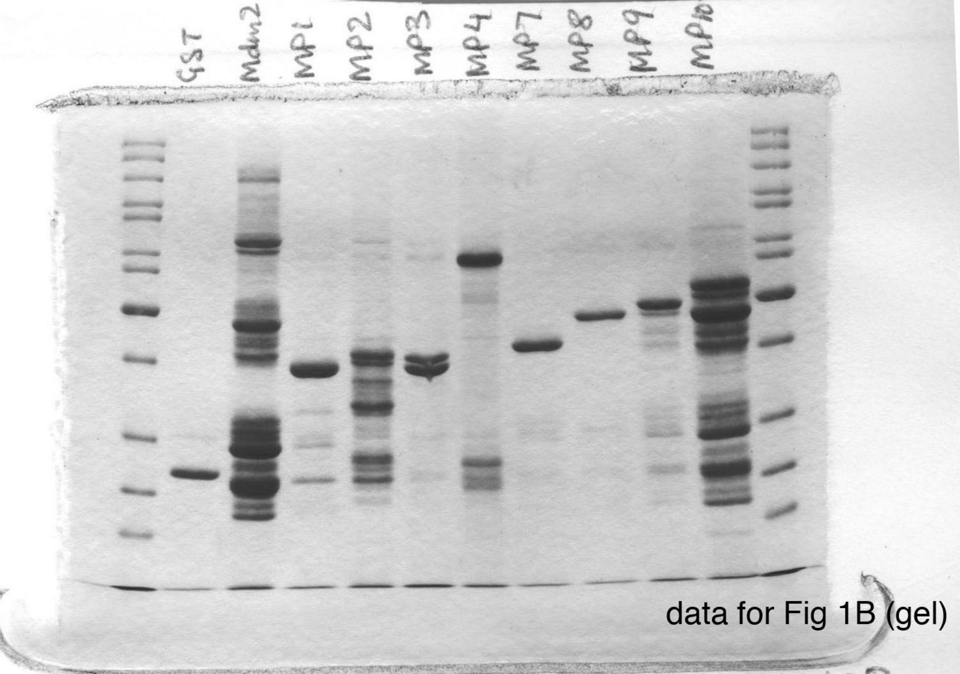

data for Fig 1B (gel)

data for Fig 2

(1)

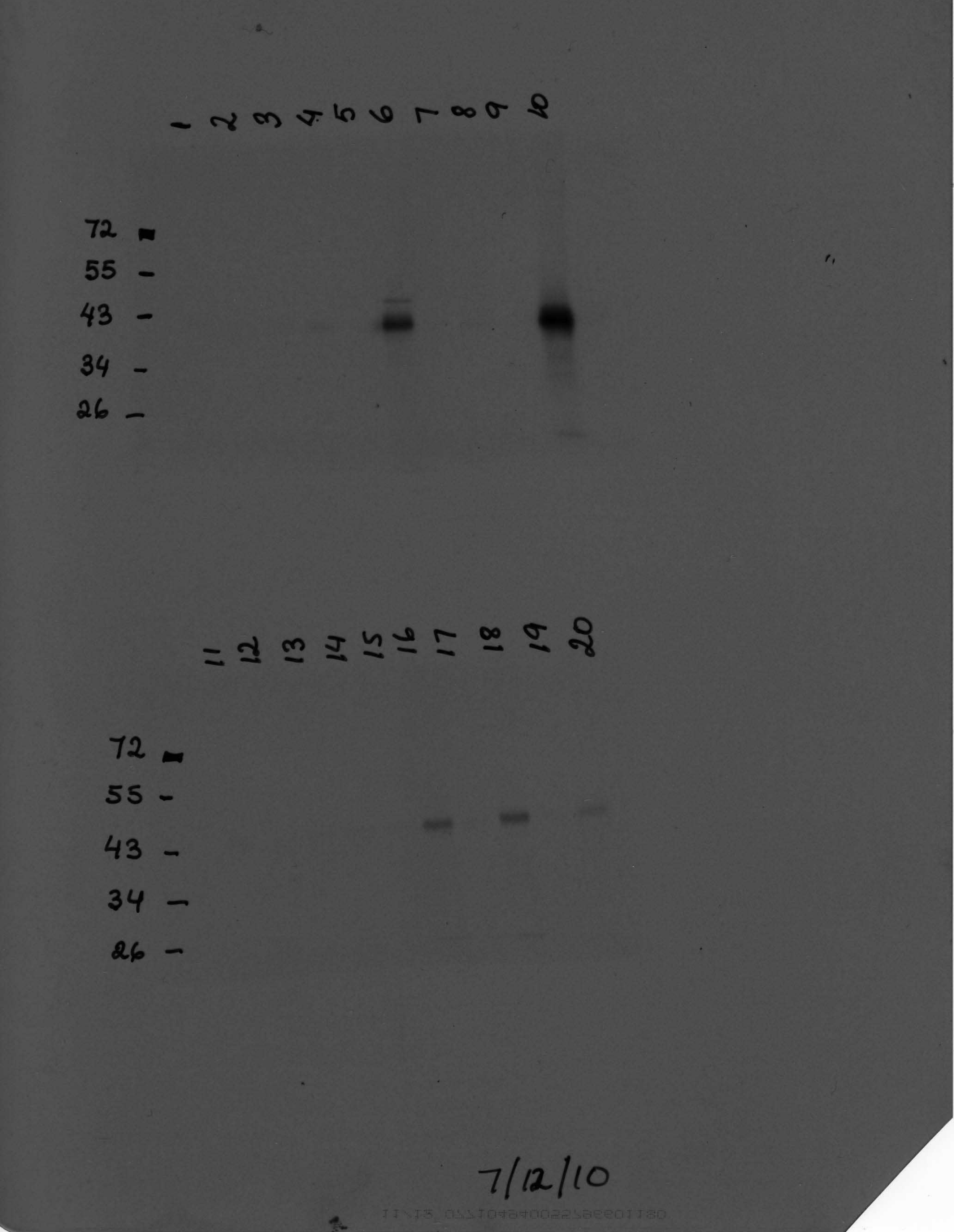

data for Fig 2 ②

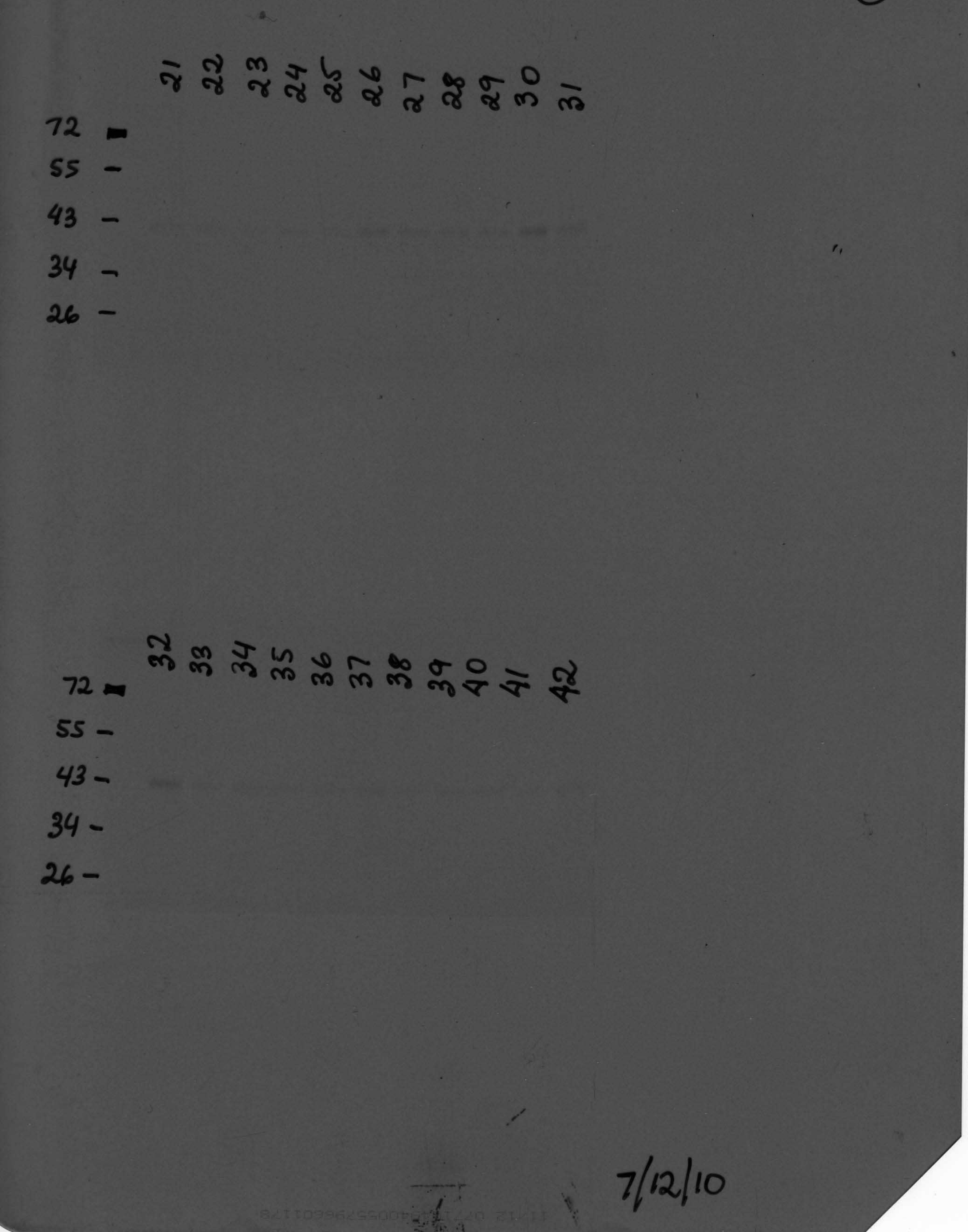

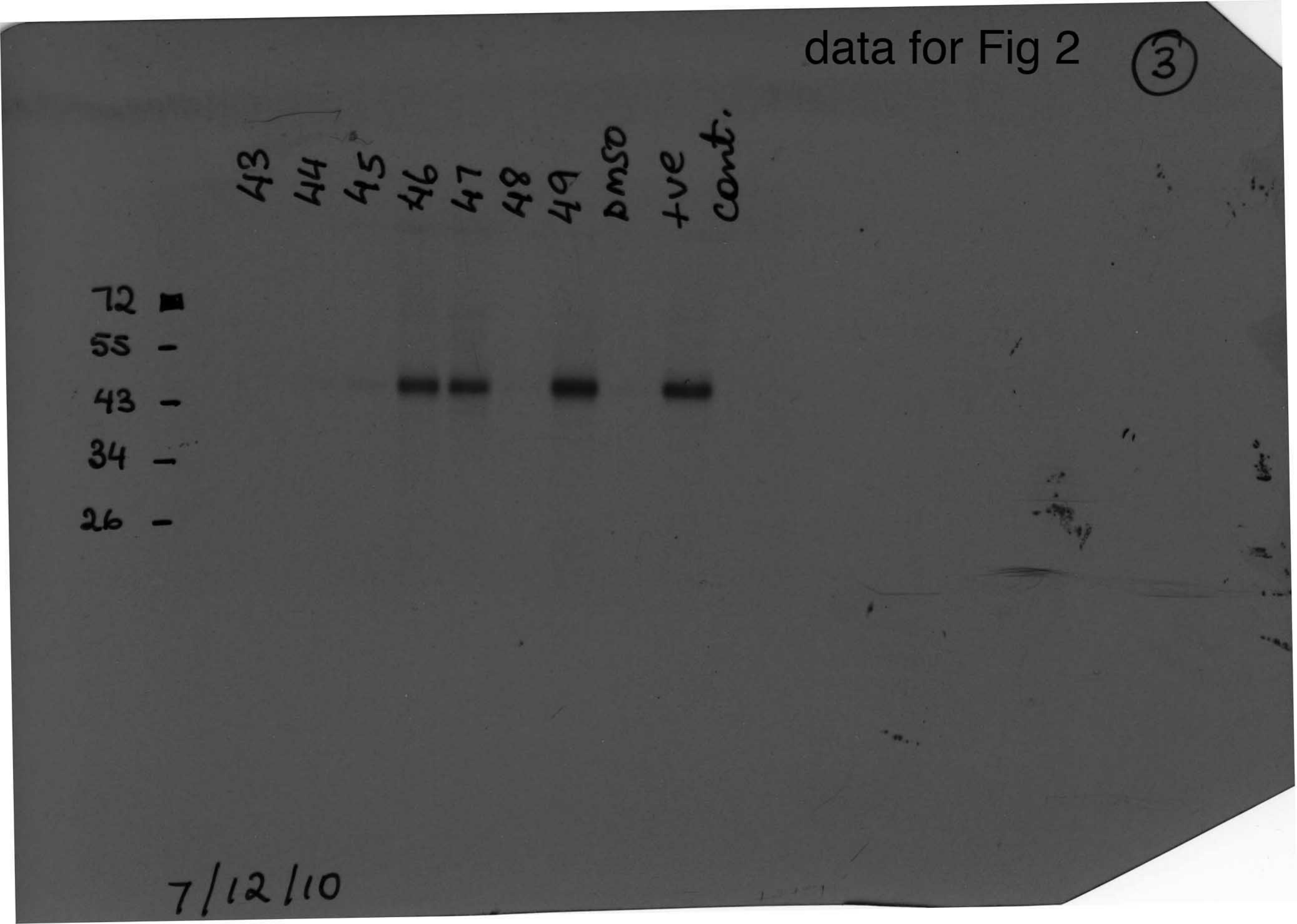
data for Fig 2
3
43
44
45
46
47
48
49
DMSO
+ve
cont.
72
55
43
34
26
7/12/10

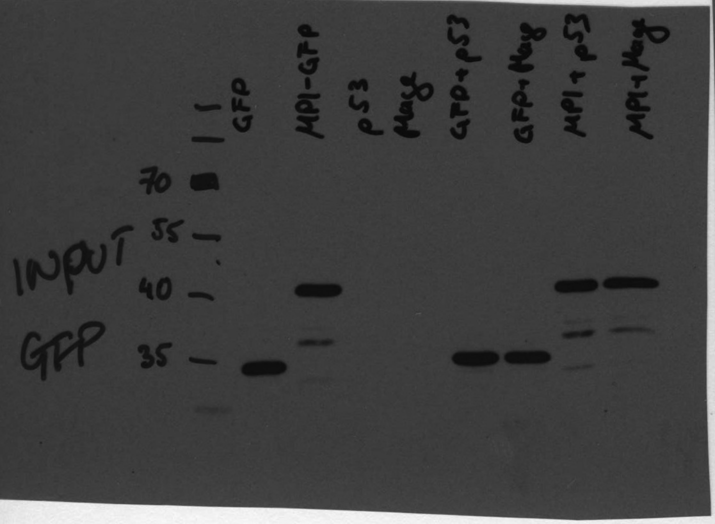

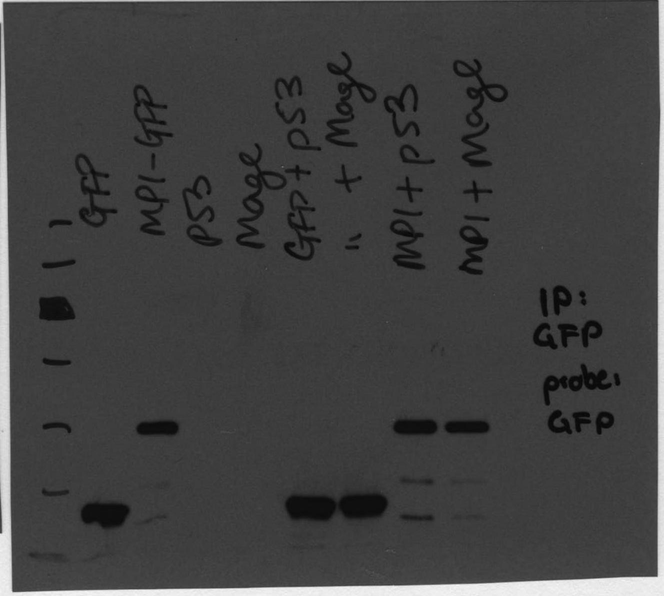

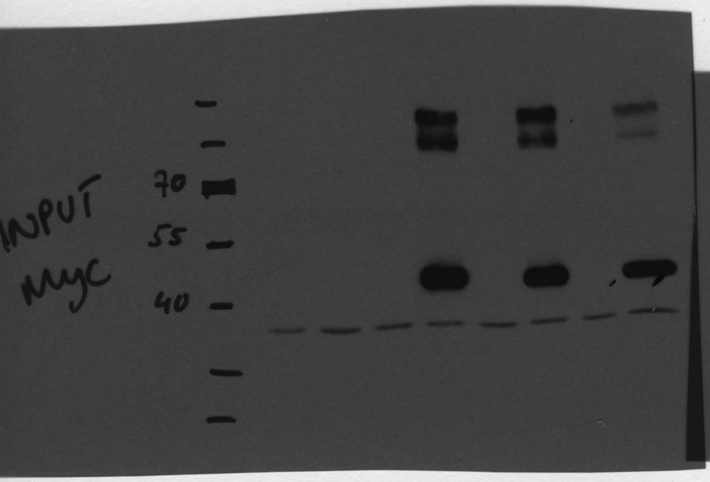

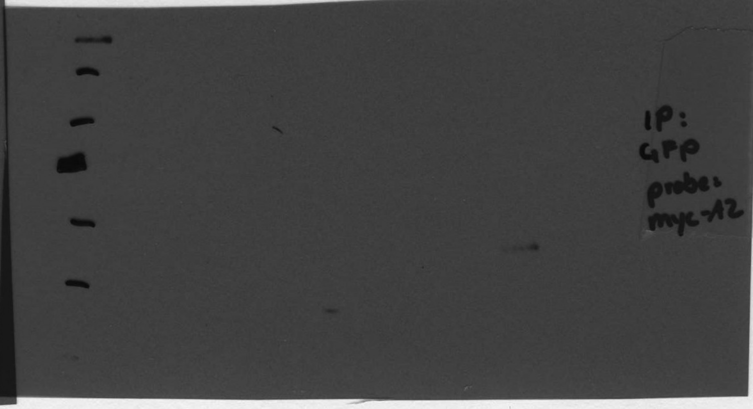

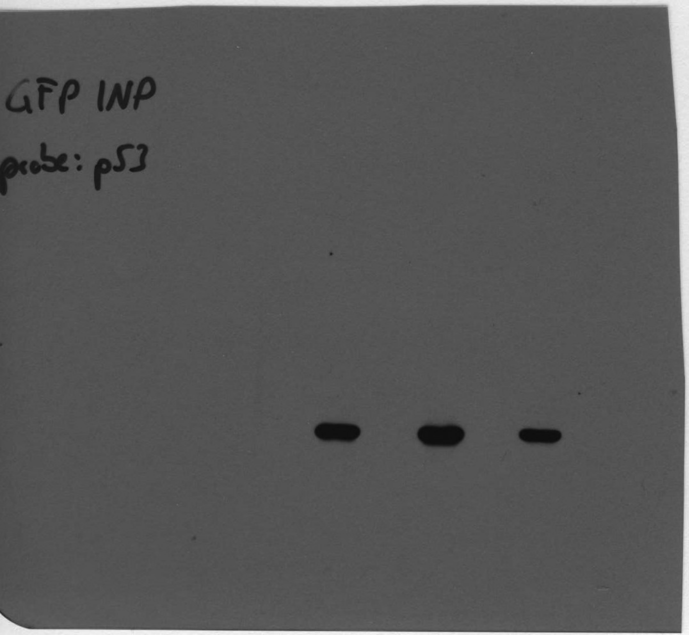

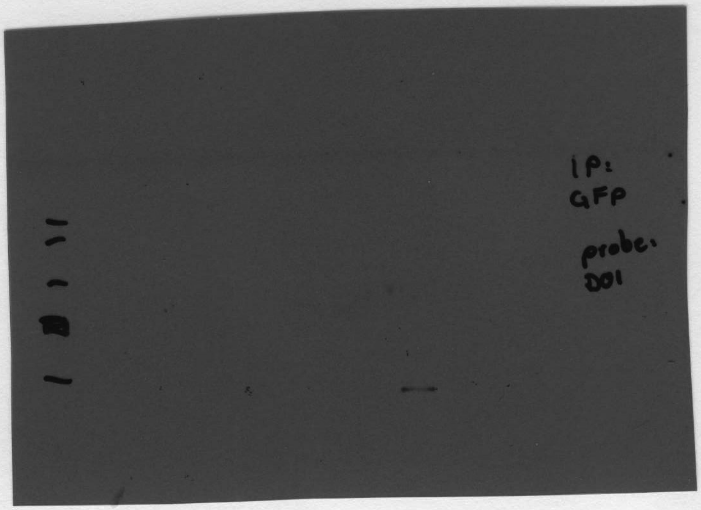

data for Fig 3C

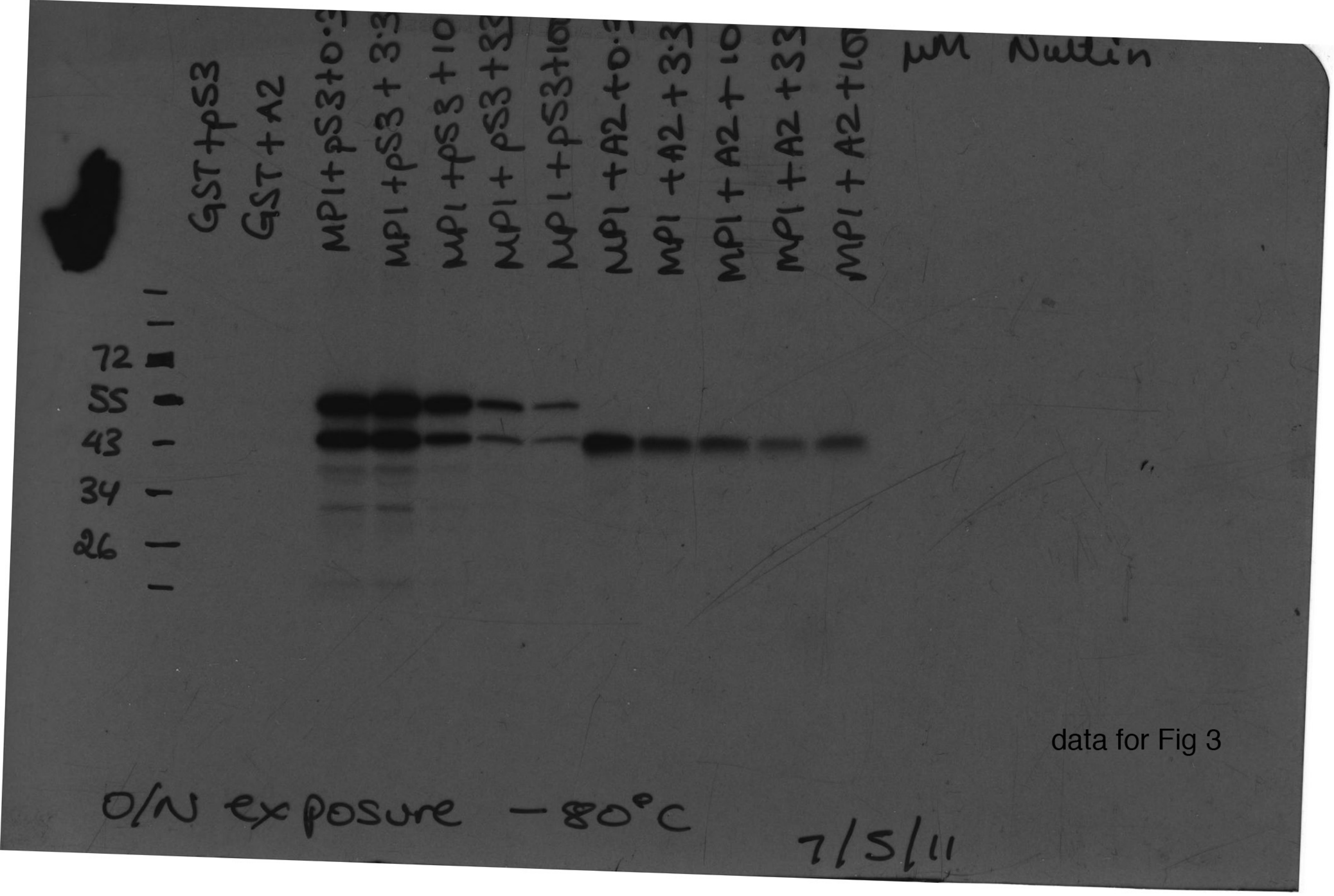

data for Fig 3

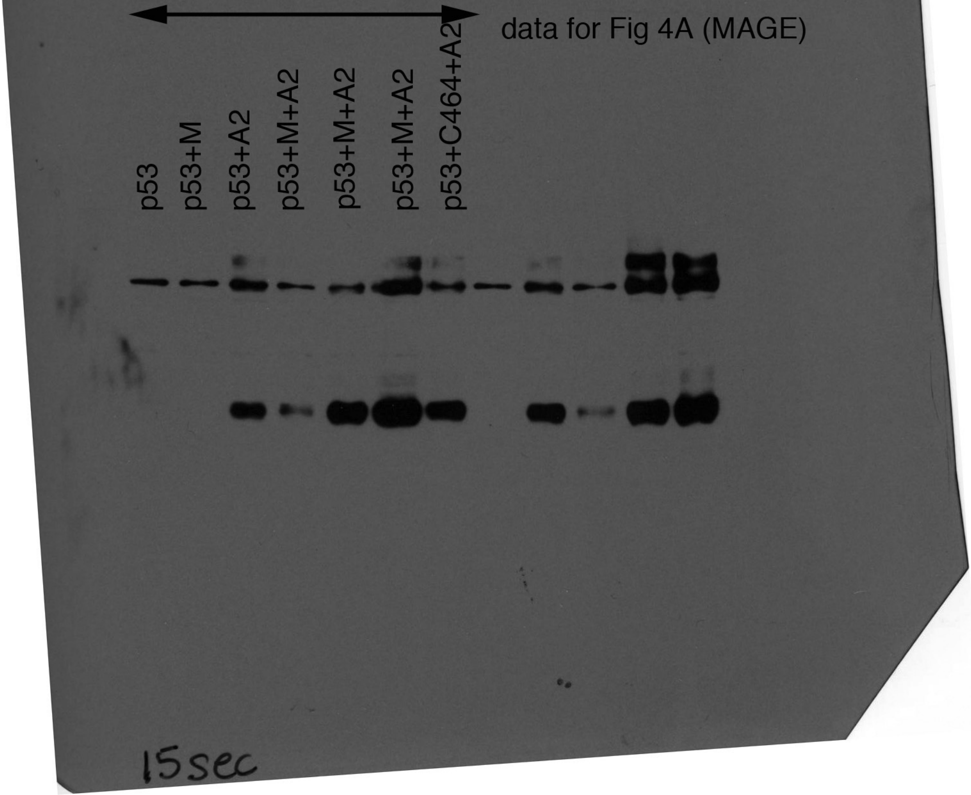
data for Fig 4A (MAGE)
p53
p53+M
p53+A2
p53+M+A2
p53+M+A2
p53+M+A2
p53+C464+A2
15sec

data for Fig 4A (MDM2)

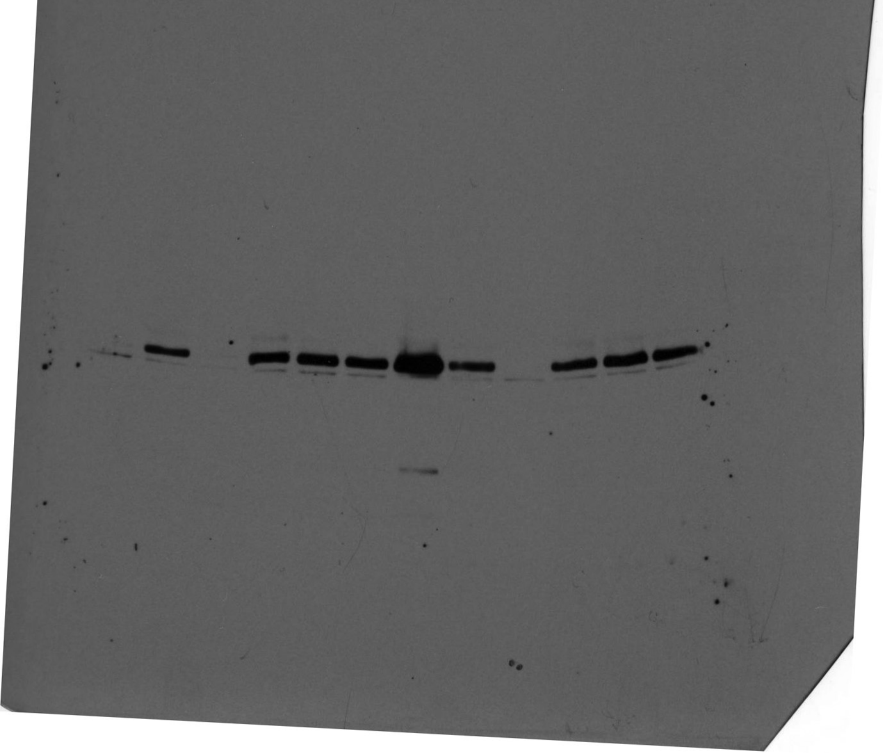

data for Fig 4A (p53)

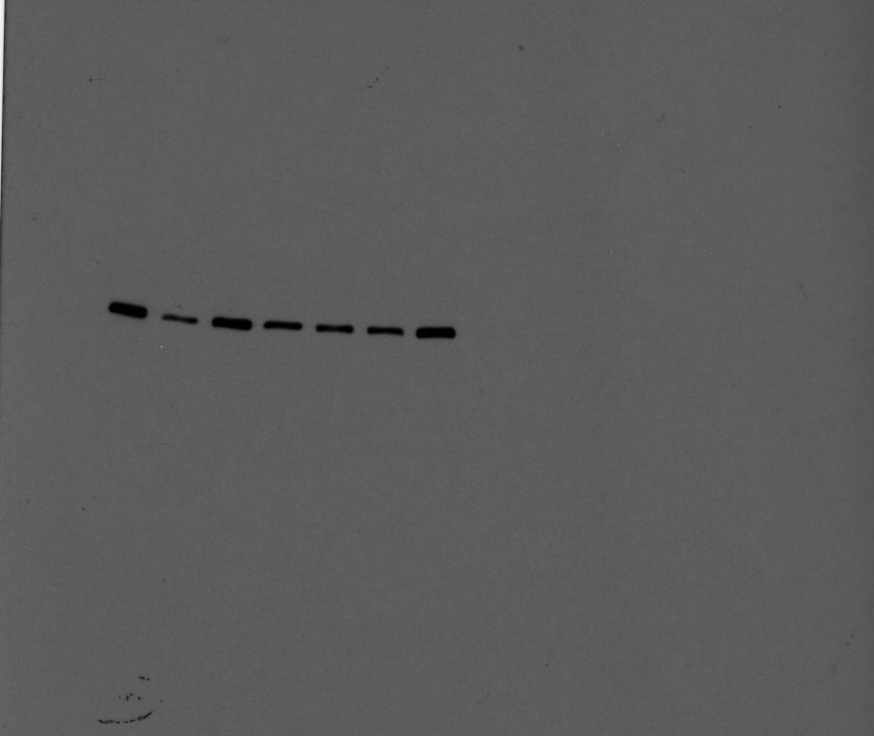

data for Fig 4A (Ub-p53)

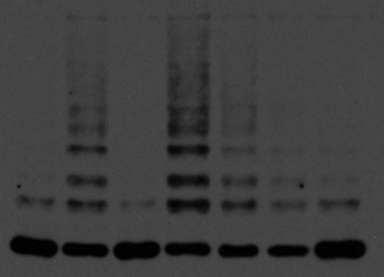

data for Fig 4A (MDM2)-Ub

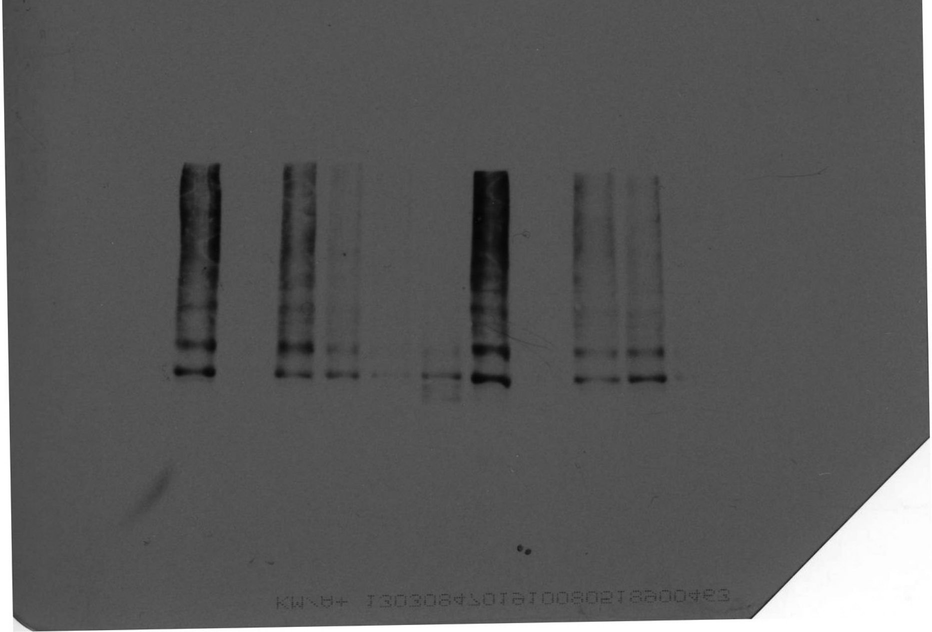

data for Fig 4A (actin)

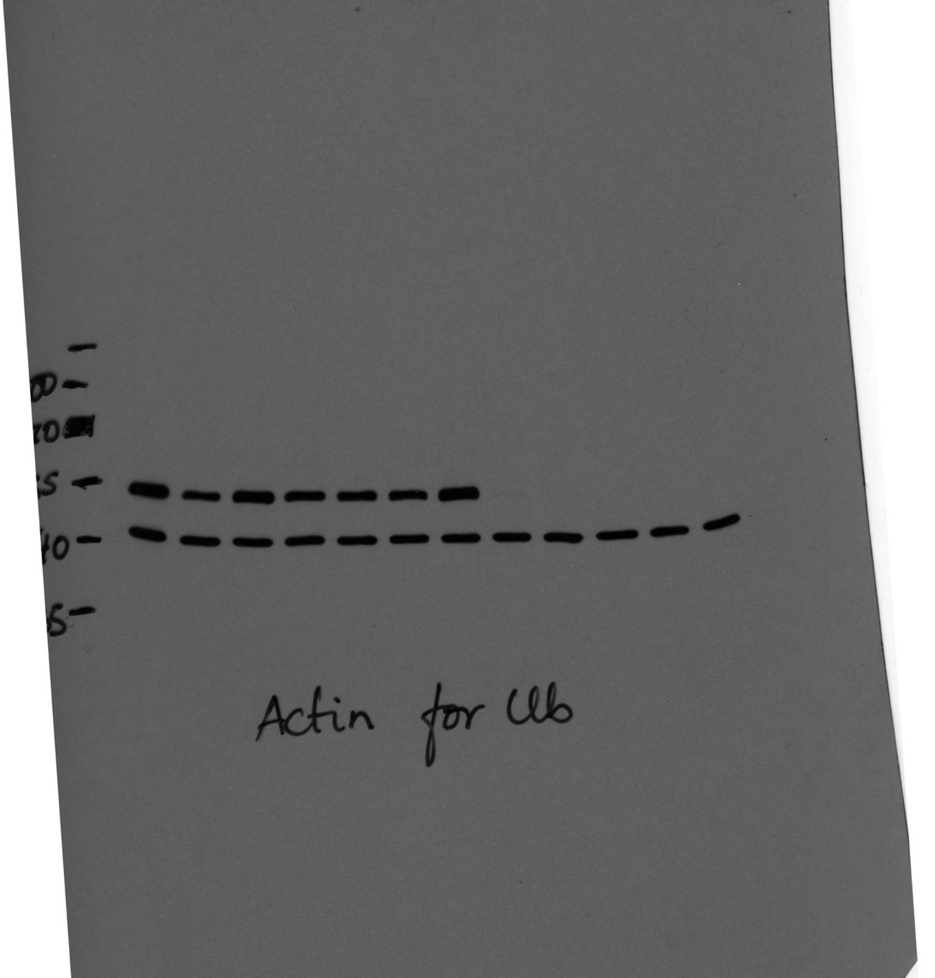

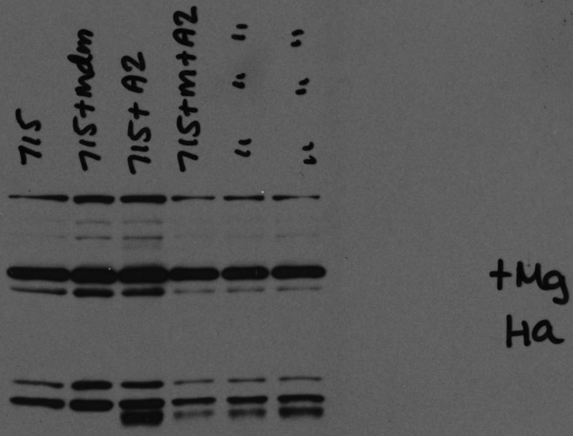

data for Fig 4B (MAGE)

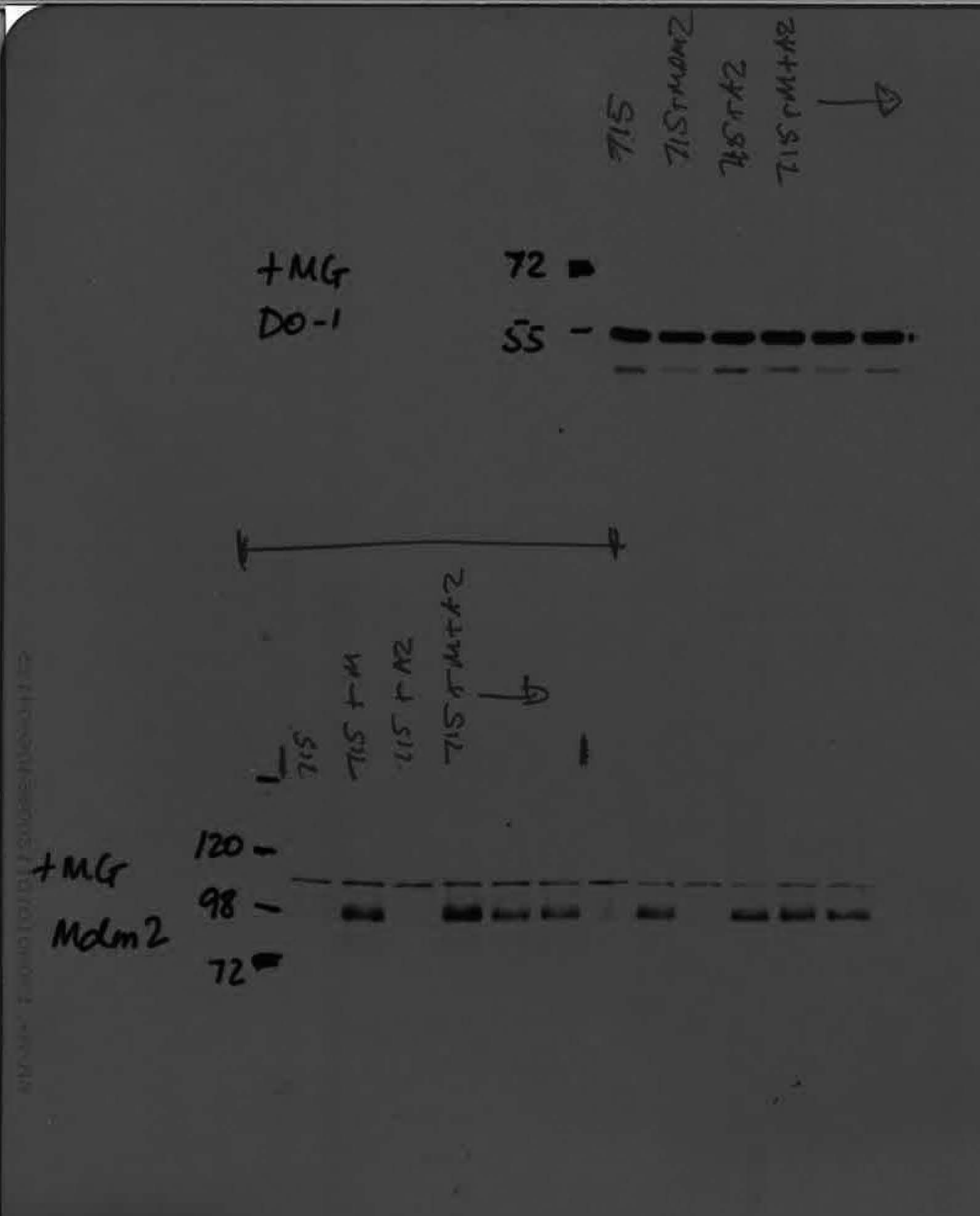

data for Fig 4B p53 and MDM2

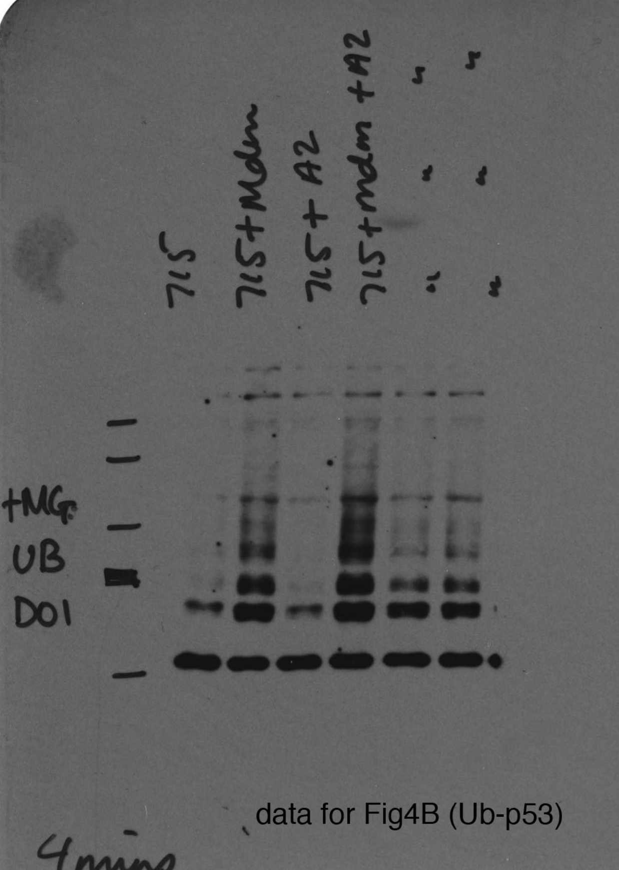

data for Fig4B (Ub-p53)

data for Fig4B (Ub-MDM2)

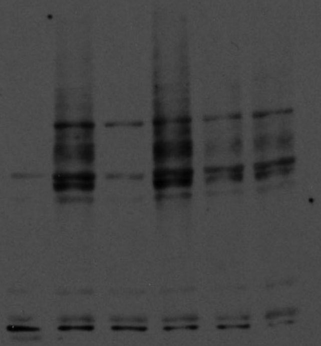

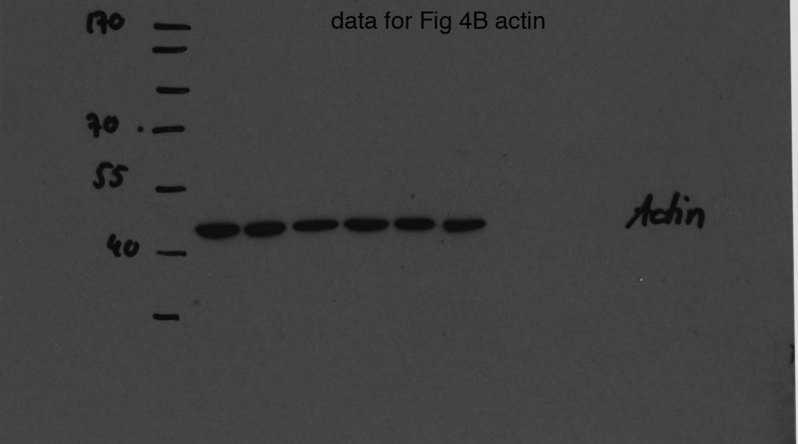

data for Fig 4B actin

data for Fig 4C

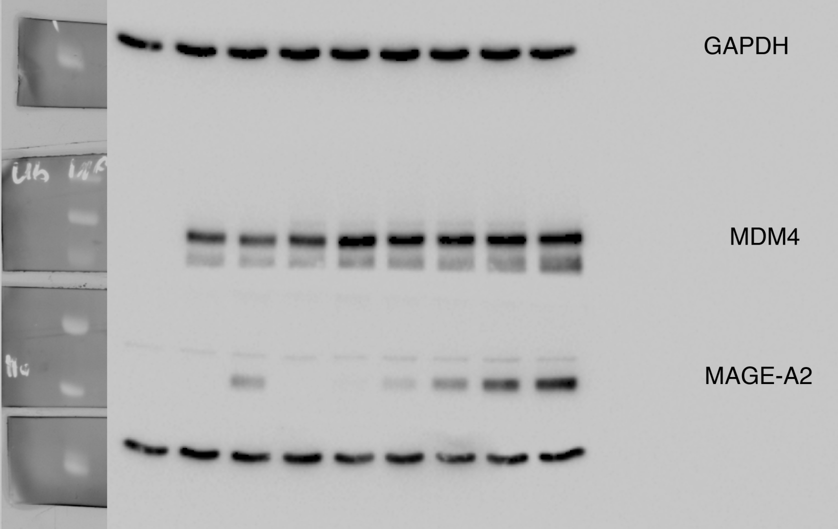

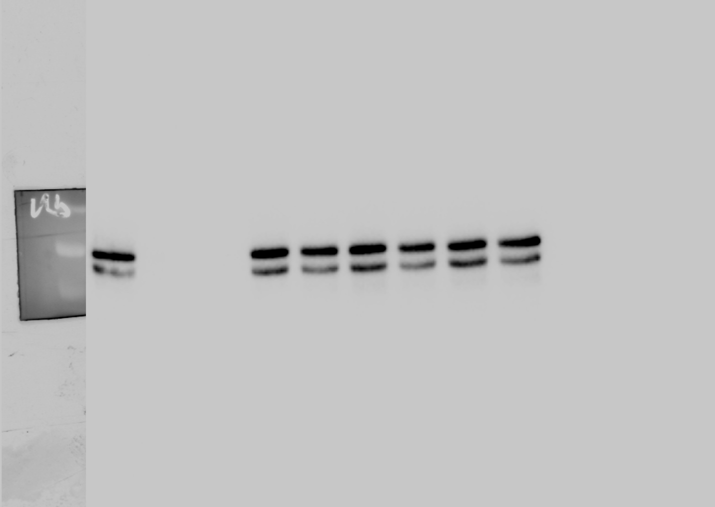

data for Fig 4C (MDM2)

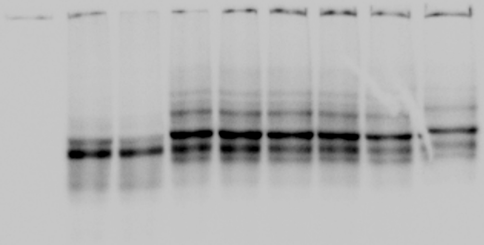

data for Fig 4C (Ub-MDM4)

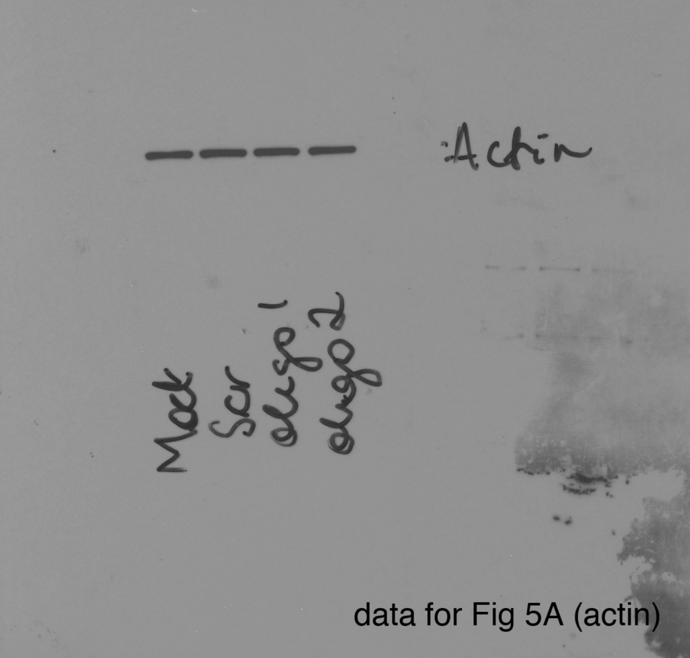

data for Fig 5A (actin)

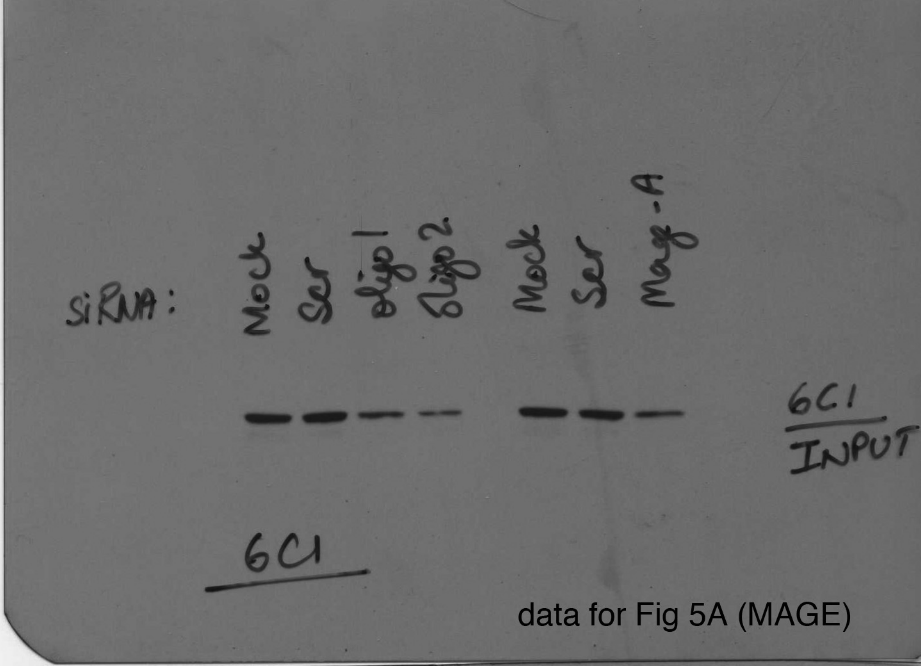

data for Fig 5A (MAGE)

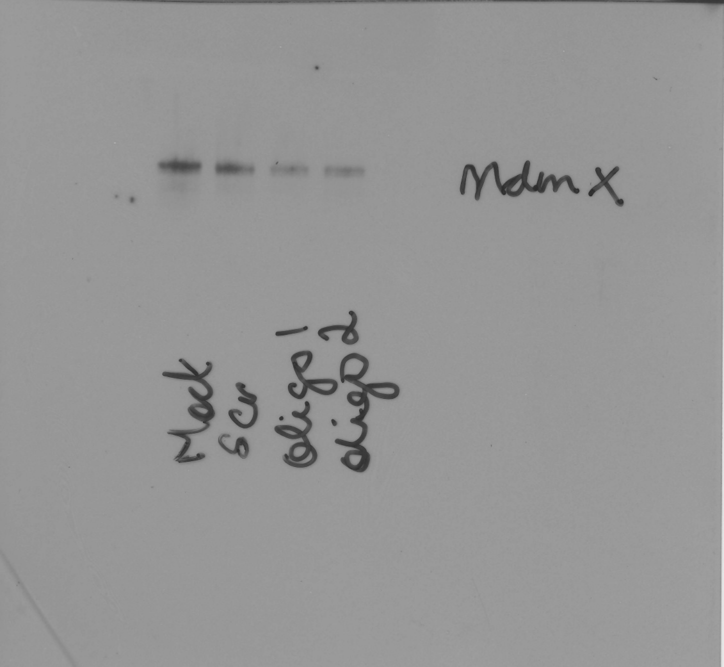

data for Fig 5A (MDM4)

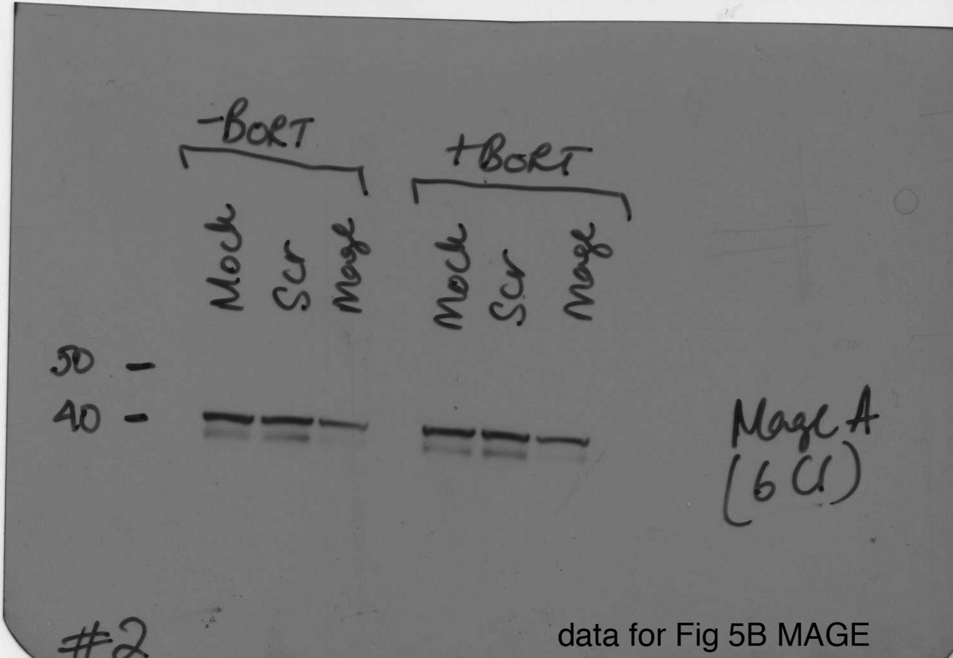

data for Fig 5B MAGE

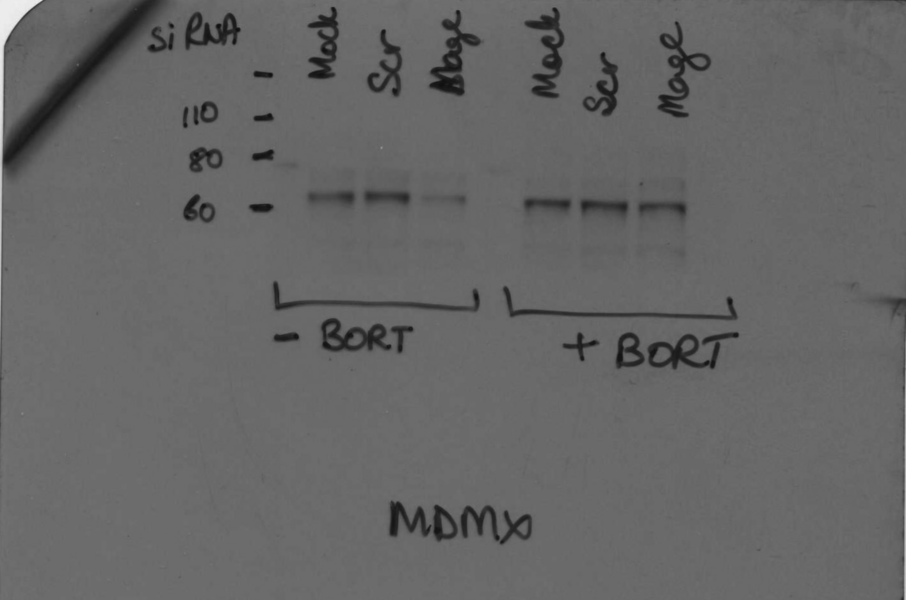

data for Fig 5B (MDM4 and actin)

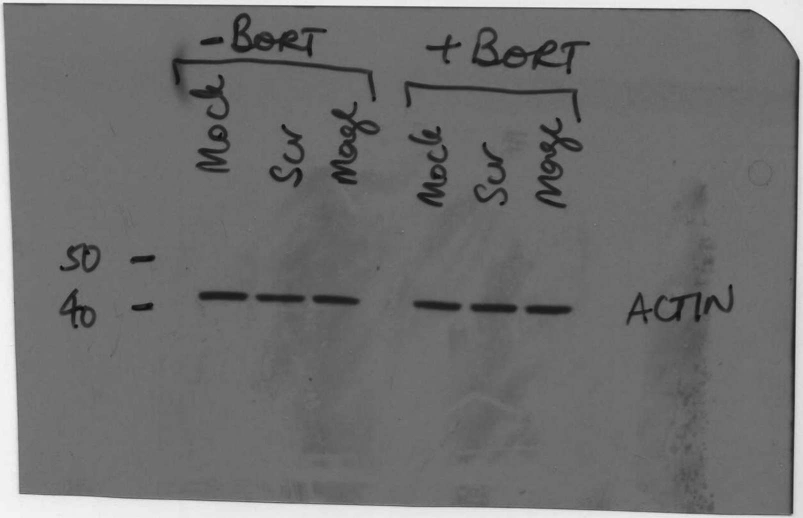

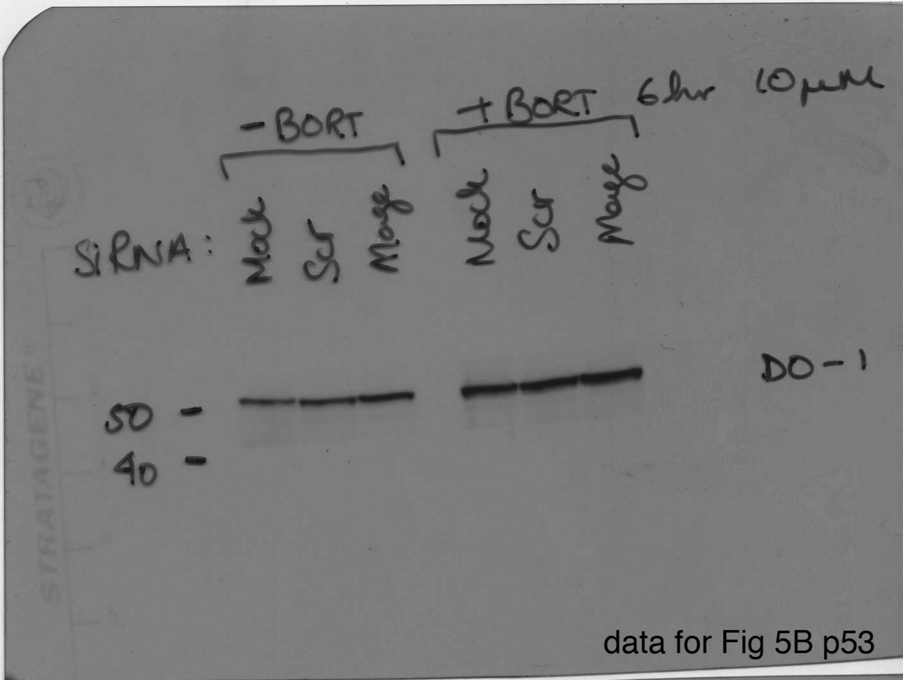

data for Fig 5B p53

data for Fig 5C actin

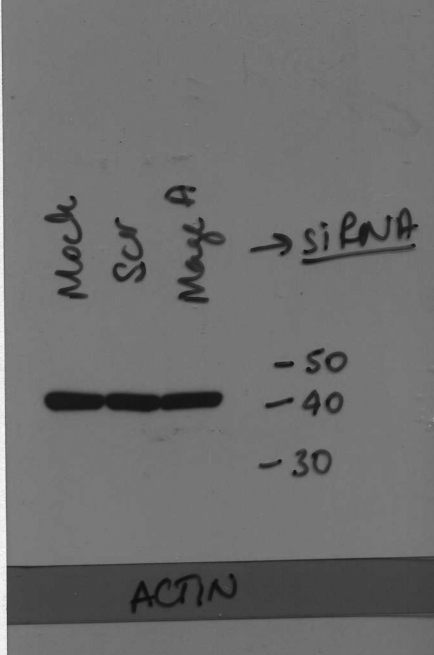

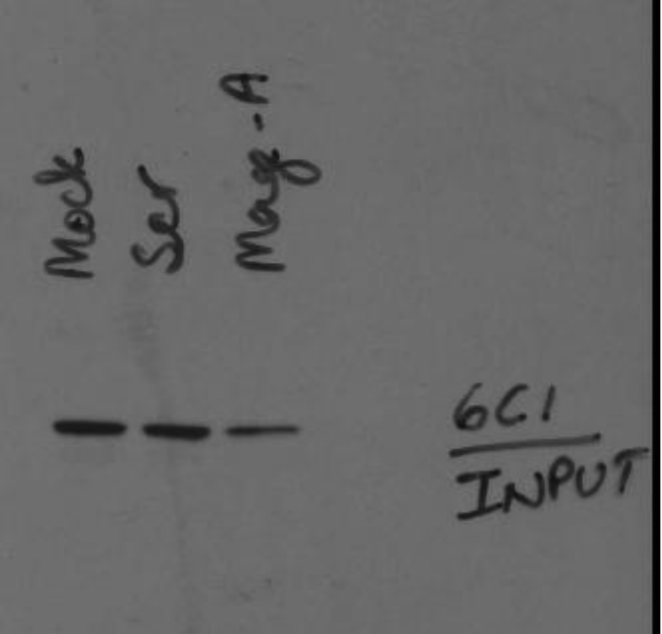

data for Fig 5C (MAGE)

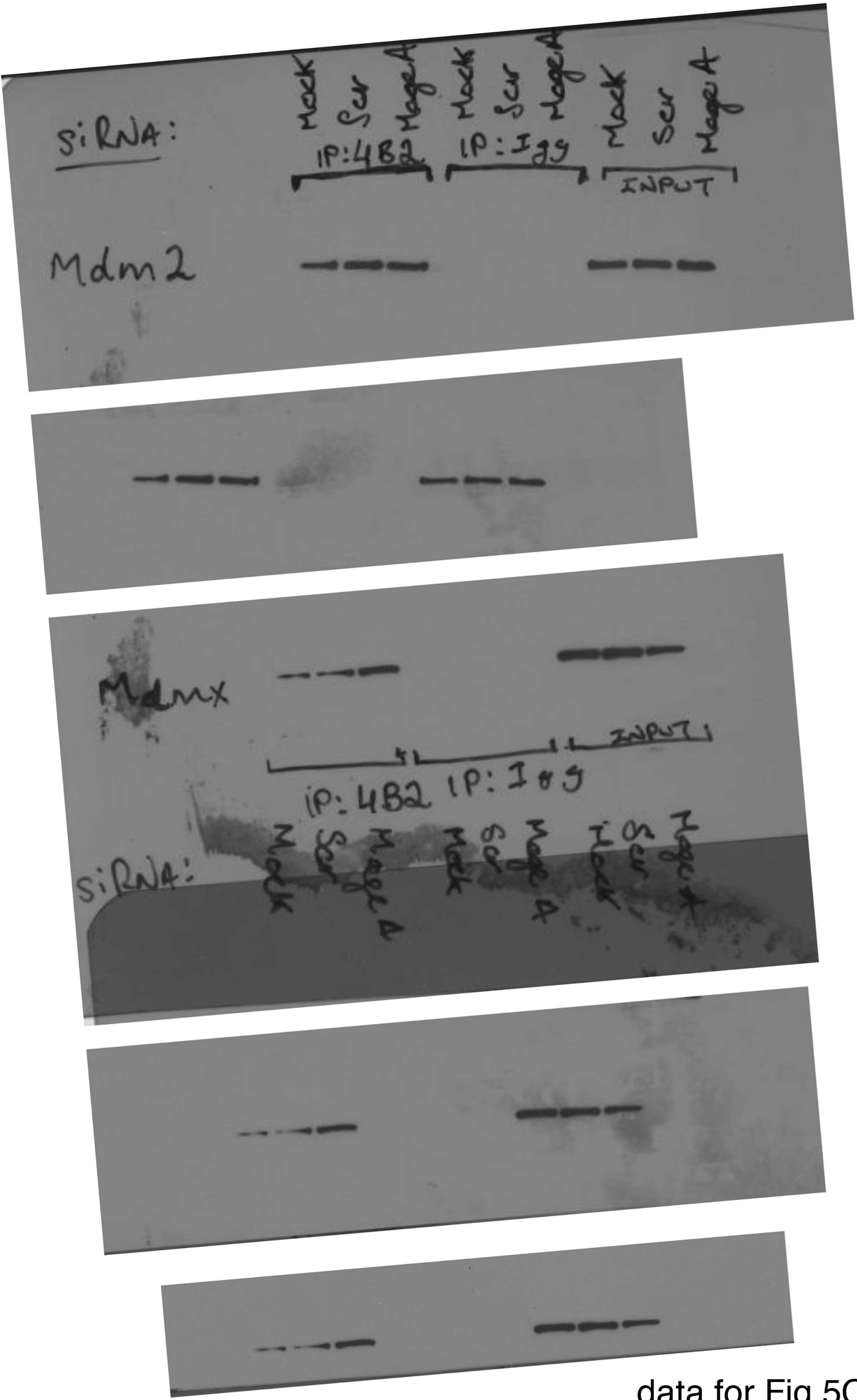

data for Fig 5C
MDM2 and MDM4(X)

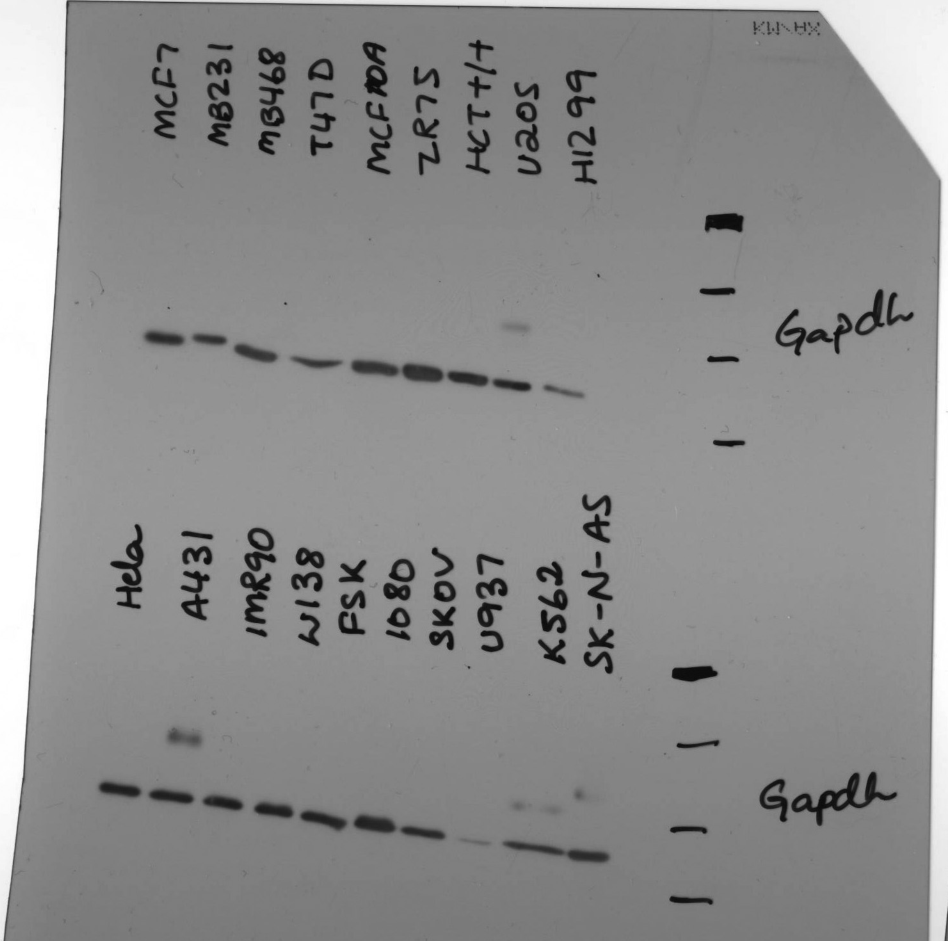

data for S1 Fig (GAPDH)

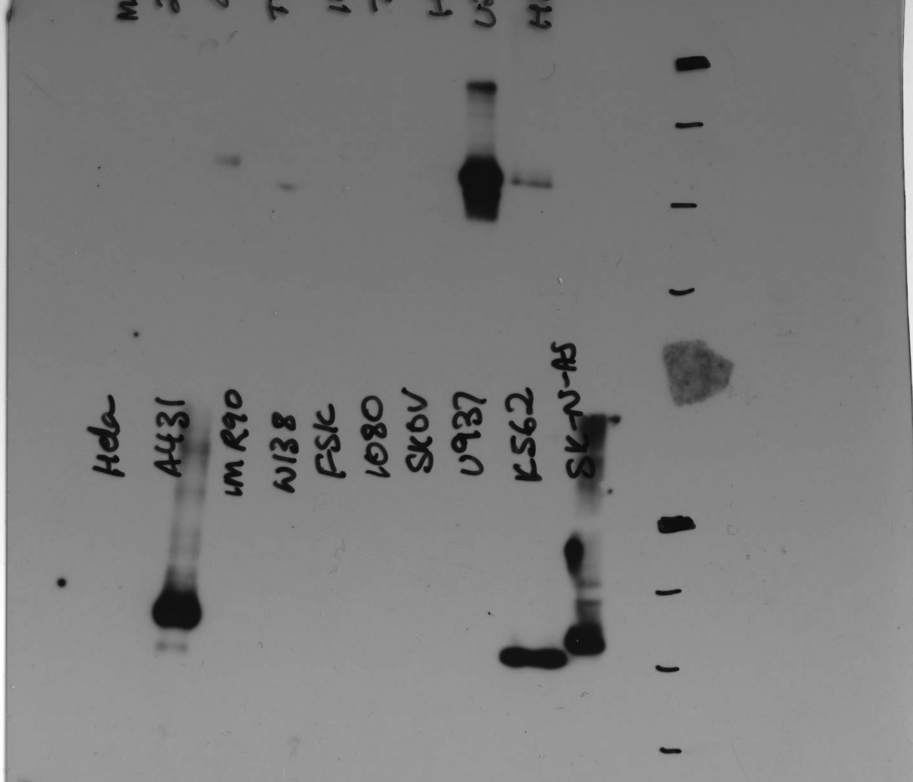

data for S1Fig (MAGE)

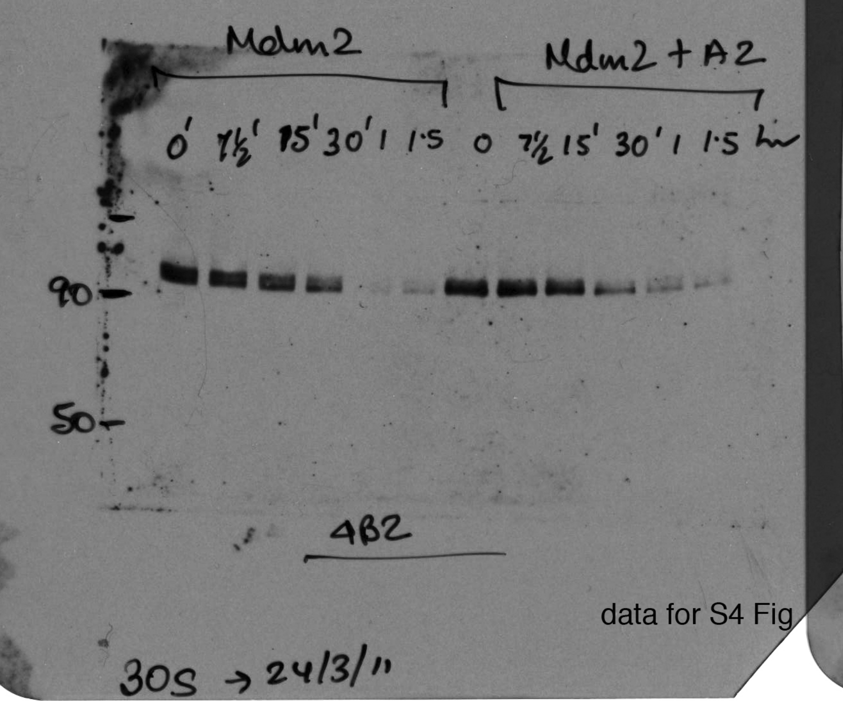

data for S4 Fig

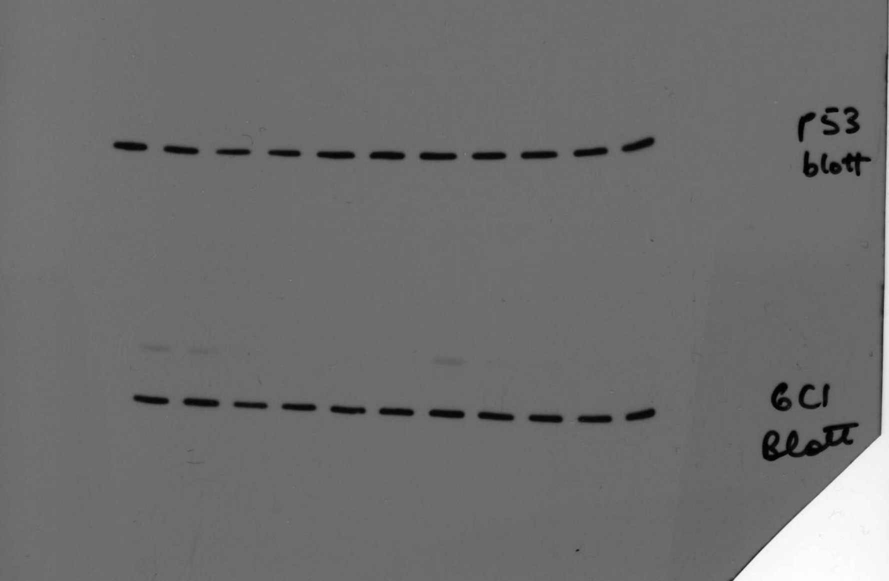

data for S5 Fig (GAPDH)

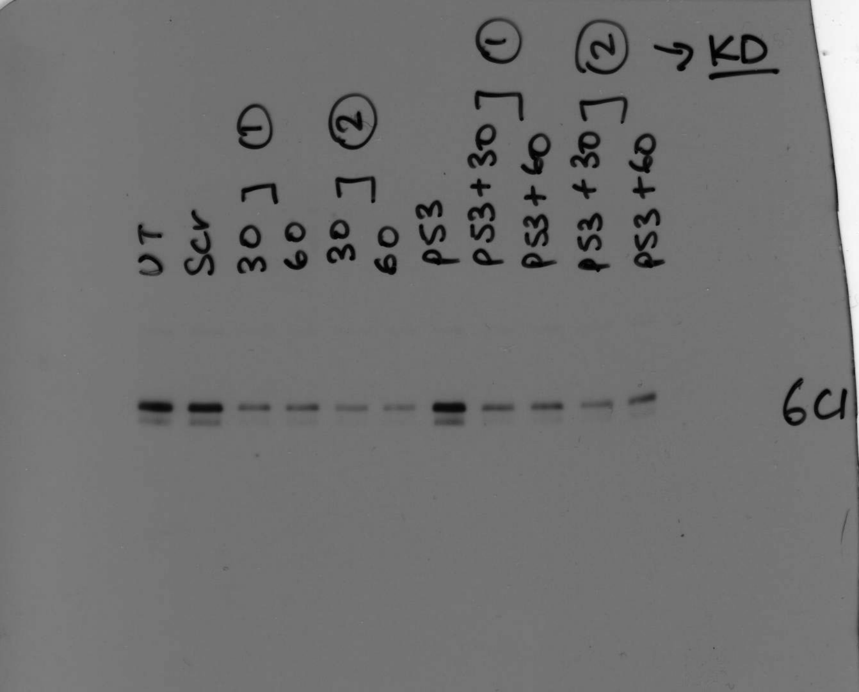

data for S5 Fig (MAGE)

SiRNA

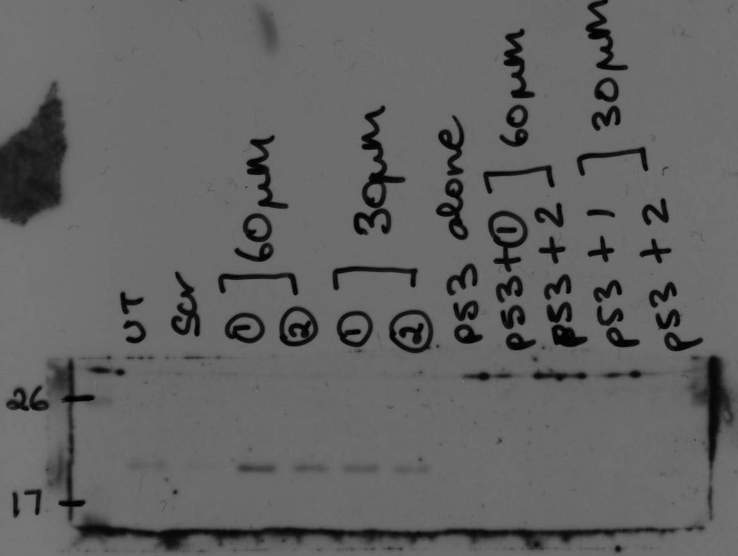

p21

5mins - 10/3/9

data for S5 Fig p21

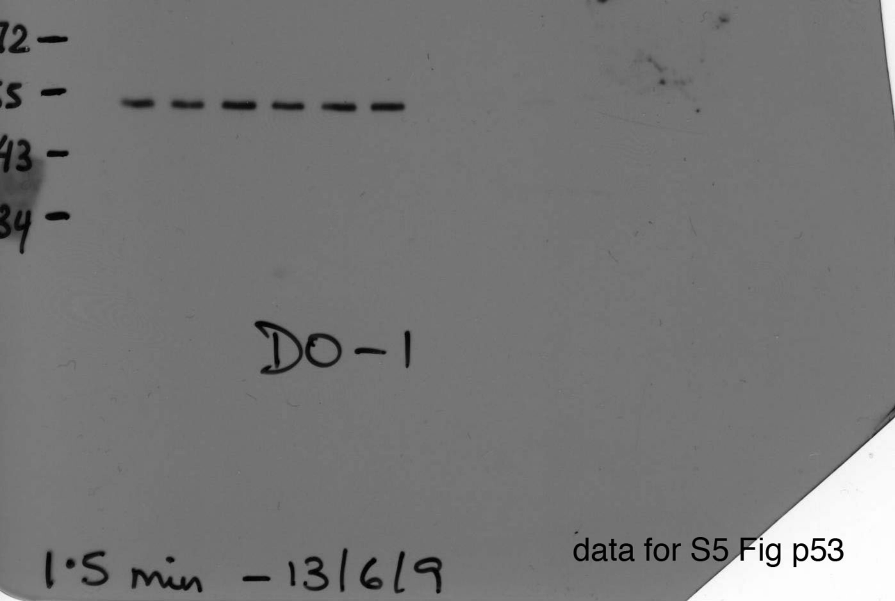

data for S5 Fig p53
